# Microscopic, Spectroscopic, and Electrochemical Characterization of Novel Semicrystalline Poly(3-hexylthiophene)-Based Dendritic Star Copolymer

**DOI:** 10.3390/polym14204400

**Published:** 2022-10-18

**Authors:** Anne L. Djoumessi Yonkeu, Miranda M. Ndipingwi, Oluwakemi O. Tovide, Morongwa E. Ramoroka, Chinwe Ikpo, Emmanuel I. Iwuoha

**Affiliations:** SensorLab, Chemical Sciences Building, University of the Western Cape, Robert Sobukwe Road, Bellville, Cape Town 7535, South Africa

**Keywords:** dendrimer, oxidative copolymerization, organic photovoltaics, semiconductor, star copolymer

## Abstract

In this study, electron-donating semicrystalline generation 1 poly(propylene thiophenoimine)-co-poly(3-hexylthiophene) star copolymer, G1PPT-co-P3HT was chemically prepared for the first time. Copolymerization was achieved with high molecular weight via facile green oxidative reaction. ^1^H NMR analyses of the star copolymer demonstrated the presence of 84% regioregular (rr) head-to-tail (HT) P3HT, which accounts for the molecular ordering in some grain regions in the macromolecule’s morphology, as revealed by the high-resolution scanning electron microscopy (HRSEM) and Selected Area Electron Diffraction (SAED) images, and X-ray diffraction spectroscopy (XRD) measurements. The star copolymer also exhibited good absorption properties in the ultraviolet-visible (UV-Vis) and the near infrared (NIR) spectral regions, which give rise to an optical energy bandgap value as low as 1.43 eV. A HOMO energy level at −5.53 eV, which is below the air-oxidation threshold, was obtained by cyclic voltammetry (CV). Electrochemical impedance spectroscopy (EIS) ascertained the semiconducting properties of the macromolecule, which is characterized by a charge transfer resistance, *R_ct_*, value of 3.57 kΩ and a Bode plot-phase angle value of 75°. The combination of the EIS properties of G1PPT-co-P3HT and its highly electron-donating capability in bulk heterojunction (BHJ) active layer containing a perylene derivative, as demonstrated by photoluminescence quenching coupled to the observed Förster Resonance charge transfer, suggests its suitability as an electron-donor material for optoelectronic and photovoltaic devices.

## 1. Introduction

The past recent years have seen an increasing number of studies centered around the development of new organic materials for optoelectronic and photovoltaic applications [1,2,3] resulting in a rapid expansion of the field with numerous compounds being produced at a tremendously fast rate [4,5,6,7]. Four main categories can be distinguished when considering the different materials that have been designed, developed, and/or investigated, namely, conjugated polymers, dendrimers, oligomers, and small dye molecules [8]. ‘Dendrimers’ form a very interesting class of molecules [9,10], which have shown good applicability in organic optoelectronic applications [11]. Similar to polymers, they consist of smaller repeating subunits, with the difference that instead of generating linear chains, the subunit grows out in a well-defined pattern (branches) from a main point. The synthesis of dendrimers has been reported to achieve (macro)molecules with high regularity and controlled molecular weight via either convergent or divergent methods [12], therefore generating non-linear and covalent structures of this class of polymers that can be accurately controlled and inducing a broad range of studies [10]. Within the ‘dendrimers’ group, π-conjugated dendrimers have been extensively investigated in organic light-emitting diodes and have been shown to be efficient charge transporters mainly due to the high-quality of films formed by them [13]. They have also demonstrated good potential applications in organic photovoltaic devices (OPVs), organic field-effect transistors (OFETs), and non-linear photonics [14]. The strong co-facial π-π interactions within these molecules allow for a high degree of molecular ordering. The monodisperse nature of dendrimers helps their film morphology, which in turn provides them with a potential advantage over conventional polymers [15]. Indeed, polymers suffer from the difficulty of achieving low polydispersity index during their synthesis which is a big drawback during device fabrication as the charge-carrier transport (mobility) within macromolecules strongly depends on their molecular weight [16,17]. The purification of these materials is easily achieved through simple processes such as column chromatography or filtration. These shape-persistent macromolecular materials with defined monodisperse structures therefore encompass the advantages of increased molecular weight of polymers with the chemical defined structures of oligomers [18]. Elements typically found in the structures of π-conjugated dendrimers include phenylenes [19], phenylene–ethynylenes [20,21], phenylene–vinylenes [22,23] carbazoles [24,25,26], truxenes [27,28], and thiophenes [29,30,31]. Among all materials, thiophene-based polymers, especially alkylated-polythiophenes, exhibit excellent charge transport properties [32]. Poly(3-hexylthiophene), (P3HT) have specifically been identified as the best organic electron-donating material due to its regioregularity, that leads to better crystallinity and higher charge-carrier mobility [33], and have been extensively studied for OPV applications [17,33,34,35]. This paper thus focuses on the first-time chemical preparation and characterization of a dendritic star-copolymer, G1PPT-co-P3HT consisting of generation 1 poly(propylene thiophenoimine), as the core of the macromolecule with poly(3-hexylthiophene) branches as the dendrons, and its spectroscopic, microscopic, and voltammetric characterizations. The potential of G1PPT-co-P3HT to be used as an electron-donating material for OPVs is also investigated.

## 2. Experimental Section

### 2.1. Synthesis

#### 2.1.1. Generation 1 Poly(propylene Thiophenoimine) (G1PPT) 

**Scheme 1:**
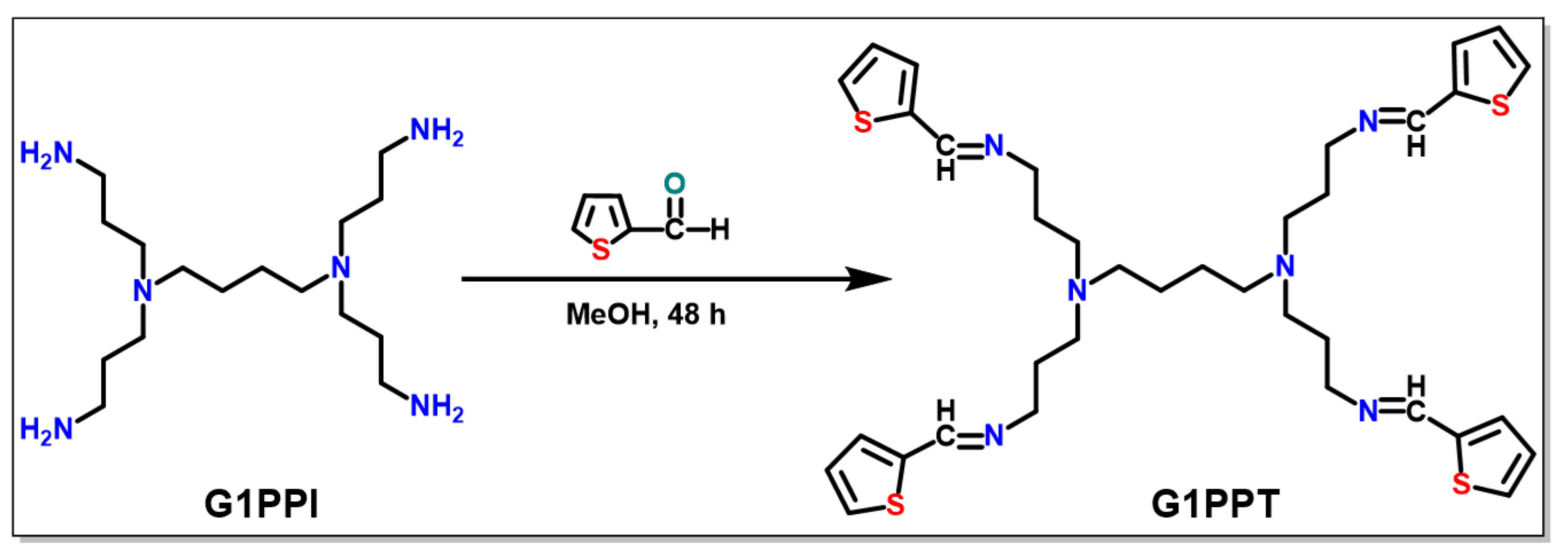
Functionalization of G1PPI to form G1PPT [1].

Functionalization of generation 1 poly(propyleneimine) tetramine dendrimer [36] (0.3863 g) using 472.55 µL (4.8821 mmol) of thiophene carbaldehyde in 25 mL methanol under N_2_ stream for 48 h that produces generation 1 poly(propylene thiophenoimine), G1PPT (Scheme 1) was confirmed by ^1^HNMR spectroscopy analysis (400 MHz) in CDCl_3_. The chemical shifts were compared to those found in the starting materials, generation 1 poly(propyleneimine) tetramine, G1PPI and 2-thiophene carbaldehyde (Figure S1). G1PPI was characterized by the following chemical shifts δ (ppm) 2.68–2.64 (t, 4H), 2.41–2.32 (2t, 16H), 1.57–1.49 (m, 12 H) and 1.33 (s, 8H) (Figure S1, Supplementary Materials). On the other hand, 2-thiophene carbaldehyde had three (3) main bands at 9.92 (s, H), 7.77–7.74 (m, 2H), and 7.25–7.18 (m, 1H) (Figure S2, Supplementary Materials). G1PPT showed a combination of the bands found in both starting materials with the difference being that the chemical shifts of the protons in the functionalized dendritic moiety are deshielded by the attachment of the thiophene ring and the formation of an imine group −N=CH, whose proton appears at 8.31 ppm. Furthermore, formation of this new bond results in a shielding effect of the thiophene protons-b and -d. Initially appearing as multiplet in 2-thiophene carbaldehyde, they have been split due to cis-trans conformation, with proton-d appearing more downfield than proton-b (Figure S3, Supplementary Materials).

#### 2.1.2. Oxidative Copolymerization of G1PPT to 3-Hexylthiophene (3-HT)

**Scheme 2:**
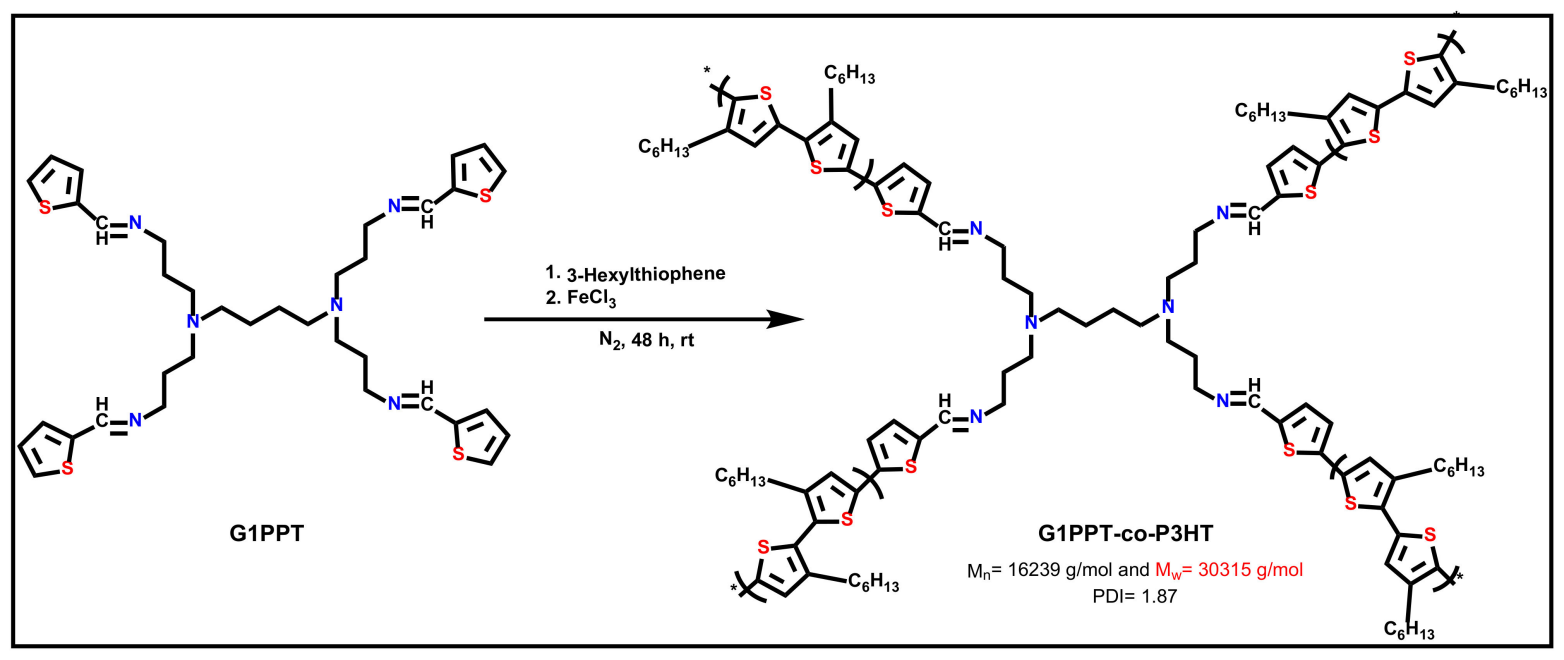
Copolymerization of G1PPT and 3-hexylthiophene in the presence of FeCl_3_ in chloroform [1].

Copolymerization of G1PPT to 3-HT followed the procedure reported by Liu et al. [37] for the polymerization of 3-hexylthiophene (Scheme 2). Specifically, 77.8 mg of G1PPT and 291 mg of FeCl_3_ were put in a 2-neck round bottom flask containing chloroform (CHCl_3_). 3-hexylthiophene (81 µL) was added dropwise to the magnetically stirred suspension and the reaction mixture was stirred for 48 h in an N_2_ atmosphere. 100 mL of methanol was added to quench the copolymerization. The prepared material was successively extracted with methanol (100 mL), acetone (100 mL), and CHCl_3_ (100 mL) by column chromatography and filtration, and G1PPT-co-P3HT copolymer was collected from CHCl_3_. Number of average molecular weight, *M_n_*, and dispersity, *PDI* values were estimated from size exclusion chromatography, performed using HPLC grade dimethylformide (DMF) at a flow rate of 0.8 mL/min. M_n_ was found to be 16,239 g/mol and the average molecular weight, M_w_ 30,315 g/mol. The dispersity, *PDI* = M_w_/M_n_ is calculated to be 1.87.

#### 2.1.3. Preparation of Poly[N,N’-bis(dodecyl)perylene-3,4,9,10-tetracarboxylic Diimide-1,7-diyl-alt-9-(heptadecane-9-yl)carbazole-2,7-diyl], (PDI-co-Carbazole)

Preparation of PDI-co-Carbazole was achieved by copolymerization of 1,7-dibromo(N,N’-bis(dodecyl)peryle-ne-3,4,9,10-tetracarboxylic diimide) (PDI-2Br) to 9-(Heptadecan-9-yl)-2,7-bis(4,4,5,5-tetramethyl-1,3,2-dioxyborolan-2-yl)-9H-carbazole (Carbazole), according to the synthetic route reported by Zhou et al. [38]. Detailed information regarding this synthesis and the characterization can be found under Section S1 in Supplementary Materials.

### 2.2. Instrumentation 

For the effective investigation, characterization, and application of the prepared macromolecule, various analytical techniques were used. Gel permeation chromatography in the form of size exclusion chromatography (GPC/SEC) was performed using an Agilent 1260 Quad Pump, using HPLC grade DMF at a flow rate of 0.8 mL/min and DMAC (0.05% BHT + 0.03% LiCl) as eluent. This technique was used for determination of average molecular weight and number of average molecular weight. Single proton Nuclear Magnetic Resonance (^1^H NMR) spectroscopy was performed using Bruker Avance III HD 400 MHz Nanobay NMR spectrometer equipped with a 5 mm BBO using tetramethylsilane as internal standard, and Fourier-Transform Infra-Red (FTIR) spectroscopy was investigated on PerkinElmer model Spectrum 100 series equipment (Boston, MA, USA). Both techniques were used for structural characterization.

For optical and photo-physical investigation, ultraviolet-visible (UV-Vis) spectroscopy measurements were performed in a quartz cuvette using a Nicolet Evolution 100 UV–Visible spectrometer from ThermoElectron Corporation (London, UK) and photoluminescence (PL) spectroscopy studies were carried out on Horiba NanoLog™–TRIAX (Edison, NJ, USA), with double grating excitation and emission monochromators with a slit width of 5 nm, and on Ocean Optics equipment. 

Microscopic characterization was achieved using high resolution transmission electron microscopy (HRTEM) using Tecnai G2F2O X-Twin MAT 200 kV field emission transmission electron microscope from FEI-ThermoFischer Scientific (Eindhoven, Netherlands), scanning electron microscopy (SEM) using ZEISS ULTRA scanning electron microscope equipped with an energy dispersive spectrometer and a Tecnai G2F2O X-Twin MAT 200 kV field emission transmission electron microscope from FEI (Eindhoven, Netherlands). Small-angle X-ray scattering (SAXS) analysis for particle size determination was carried out on an Anton Paar SAXSpace system (Graz, Austria), using copper Kα radiation (0.154 nm) equipped with a 1 D Mythen 2 position sensitive detector and a beam stop alignment. The sample was scattered at a thickness of 315 mm and the sample chamber was evacuated to below 5 mbar to avoid further air scattering. X-ray diffraction patterns for the material were obtained using a D8 advance diffractometer (BRUKER-AXS) employing copper Ka radiation with a wavelength of 0.154 nm, operating at a voltage of 40 kV and current of 40 mA.

Cyclic voltammetry (CV) and electrochemical impedance spectroscopy (EIS) studies were conducted on CH instruments (Austin, TX, USA). Electrochemical measurements of G1PPT-co-P3HT drop-coated film on a working electrode were performed at ambient air in a solution of acetonitrile containing 0.1 M tetrabutylammonium hexafluorophosphate (Bu4NPF6). A three-electrode system with a gold (Au) working electrode, a Pt wire as the counter electrode and Ag/AgCl or Ag/Ag^+^ as reference electrode, was used. Additionally, for frontier orbital determination, cyclic voltammetric measurements were carried out with reference to ferrocene.

### 2.3. Thin Films and Device Fabrication

Devices were fabricated according to the following procedure, where the device configuration used was glass/indium tin oxide (ITO)/poly(3,4-ethylene dioxythiophene):poly(styrene sulphonate), (PEDOT:PSS)/active layer/Al with a pixel area of ~0.0256 cm^2^. Prior to fabrication, the patterned ITO-coated glass substrates with resistance 20 Ω/m^2^ were cleaned. The cleaning procedure was as follows: the substrates were sonicated in Hellmanex solution for 15 min and rinsed twice in boiling water; then, they were sonicated for 15 min in N, N-isopropanol, and rinsed again twice in boiling water. Afterwards, the substrates were dried under an argon (Ar) gas stream. Subsequent to the cleaning process, the hole transporting layer, PEDOT:PSS (Heraeus Clevios PH-1000), was deposited on the ITO surface by spin coating at 5000 rpm for 30 s; then dried on a hot plate at 150 °C for 5 min. PEDOT-PSS was used to reduce the roughness of the ITO layer and acts as electron blocking layer (EBL) between the ITO and the active layer. PEDOT-PSS was gently wiped away from the etched part of the ITO using de-ionized water with a cotton bud to enhance contact with the electrode. The active layer consists of an intermixed donor: acceptor bulk heterojunction blends. The prepared donor:acceptor solutions in chloroform (50 µL) were spin-coated on the PEDOT:PSS at 900 rpm for 105 s, dried on a hot plate at 80 °C for 10 min and the edges were cleaned using chloroform. Finally, the top electrode Aluminum layer (103 nm) deposition was carried out by thermal evaporation on the glass/ITO/PEDOT-PSS/BHJ active layer substrate to complete the device fabrication. A drop of encapsulation epoxy was deposited on a glass substrate and used to seal the device that was then dried under UV lamp to avoid oxidation from air contact. Current-voltage (I–V) curves of the devices were recorded in the dark and under illumination using a solar simulator. All characteristic measurements were carried out at ambient air and recorded under simulated solar illumination of AM 1.5 with an incident power density of 100 mW/cm^2^ from −0.2 to 1 V using a Keithley output reader. Although the main device of interest is based on G1PPT-co-P3HT/PDI-co-Carbazole (2:1) bulk heterojunction blend, three other devices based on G1PPT-co-P3HT: PC61BM (1:2), P3HT: PDI-co-Carbazole (2:1), and P3HT:PC61BM (2:1) were also fabricated to investigate the photovoltaic characteristics of each prepared copolymer with respect to well-known widely used P3HT and PCBM as donor and acceptor, respectively.

## 3. Results and Discussion

### 3.1. Material Characterization 

#### 3.1.1. Structural Characterization

**Figure 1:**
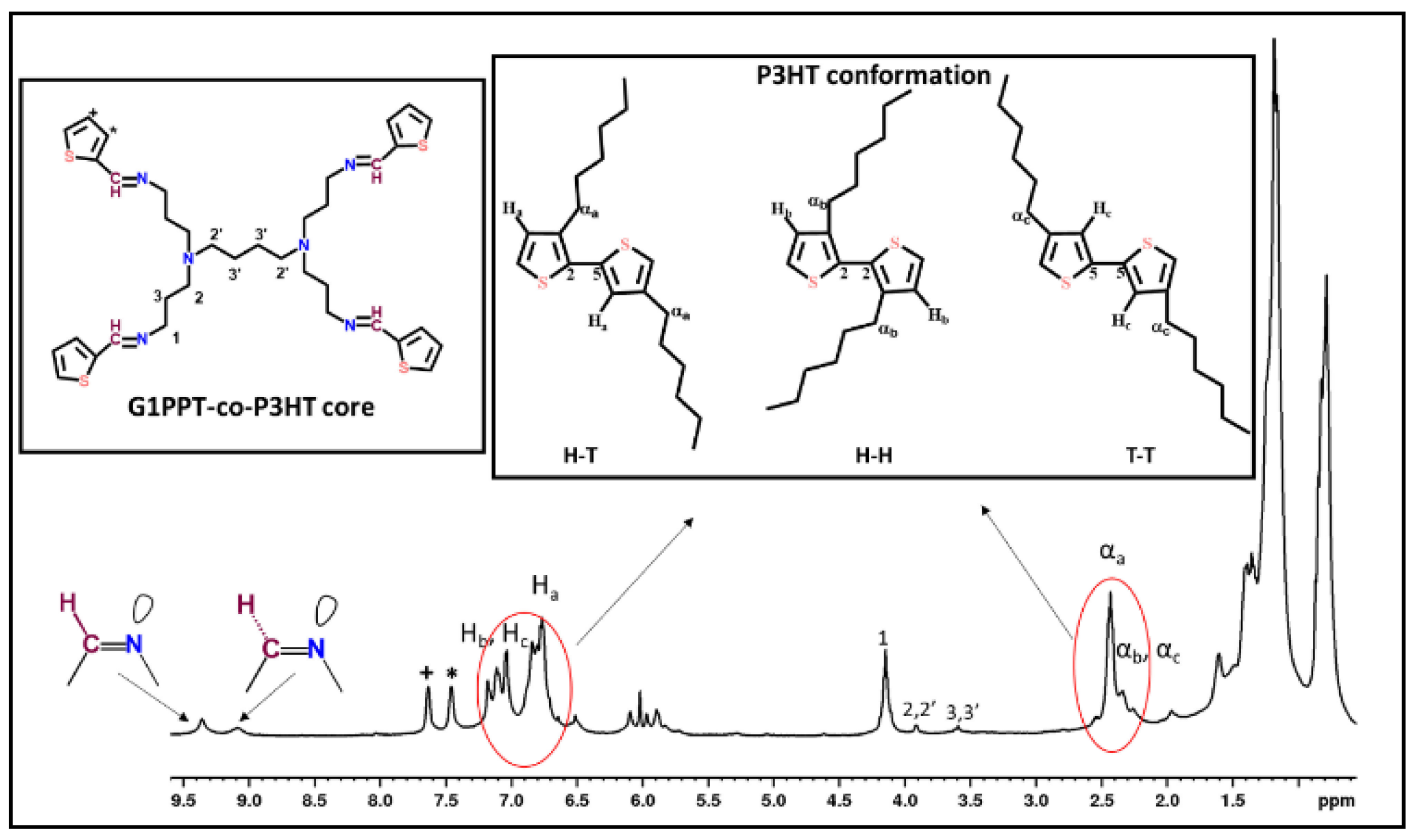
^1^H NMR of generation 1 poly(propylene thiophenoimine)-co-poly(3-hexylthiophene) [1].

**Figure 2:**
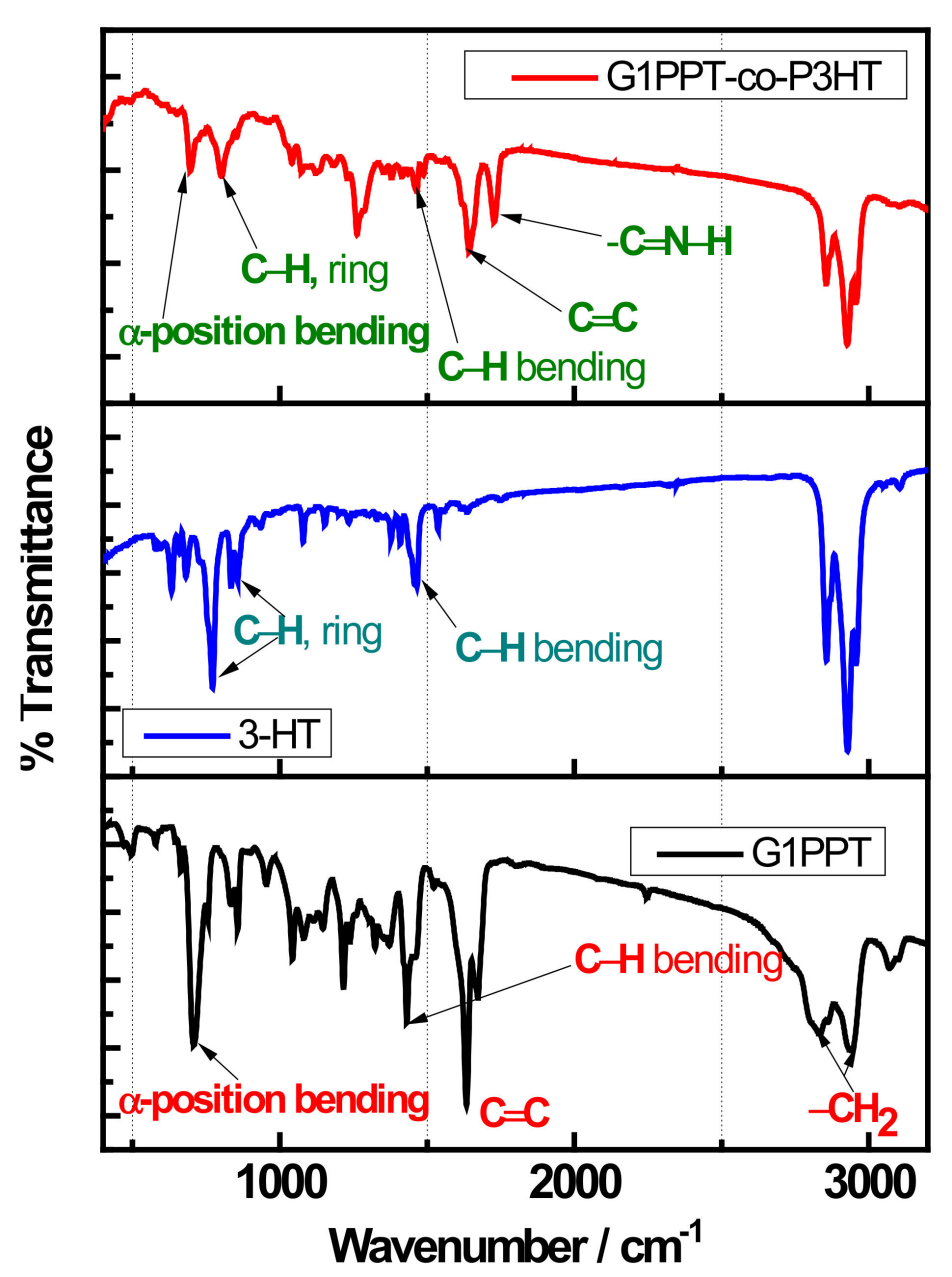
FTIR spectra of G1PPT-co-P3HT, 3-HT, and G1PPT [1].

^1^H NMR (CDCl_3_, 400 MHz) spectroscopy was used as the key technique to ascertain the structure and purity of star copolymer poly(propylene thiophenoimine)-co-poly(3-hexylthiophene) (G1PPT-co-P3HT). New signals were observed with respect to G1PPT (Figure 1). In general, extended conjugation and resonance effects resulted in deshielding the chemical shifts of most of the protons within the core of the star copolymer, compared to the bands in the functionalized dendrimer, while peaks of P3HT conjugated chain were mostly shielded compared to reported chemical shifts of regioregular poly(3-hexylpolythiophene), rr-P3HT. The small peaks appearing in the further downfield region, 9.36 and 9.09 ppm, are due to the proton in the imine group (−N=CH) which is highly deshielded in the star-copolymer compared to the functionalized dendrimer due to the effect of extended conjugation. The two peaks are suggested to be due to the cis and trans- conformations exhibited by the protons. In the region 7.8–6.7 ppm, the protons on the non-alkylated and alkylated thiophene rings are found. Here, the furthest last two singlets are due to the two protons on the non-alkylated thiophene rings while the other multiplets are from P3HT. Extended conjugation in the star-copolymer, coupled to the presence of electron-withdrawing N atoms in the imine groups, shift the peaks of the non-alkylated thiophene ring protons, a multiplet between 7.18–7.04 ppm, to appear more downfield compared to the functionalized dendrimer, G1PPT. We also suggest there could be a contribution of resonance effect. According to the literature, regioregular (rr) head-to-tail (H-T) P3HT with more than 95% regioregularity, is characterized by the thiophene unit proton appearing at 6.98 ppm [29,39,40]. In contrast, in P3HT with a lower extent of regioregularity, where there is a combination of two or all of H-T, head-to-head (H-H), tail-to-tail (T-T) configurations, more peaks are observed between 7.05–7.00 ppm [41], as it is the case from our spectrum. In the region around 4.15–3.91 ppm, the peaks are found from the protons in the –CH_2_ groups in between the tertiary amino groups. The alkyl group protons α-positioned to the thiophene rings, as presented in the P3HT conformations box within Figure 1, arise further up-field between 2.5–2.4 ppm as a multiplet, confirming the existence of H-T, H-H and T-T P3HT [40]. The other protons, i.e., the methylene groups within the core of the star copolymer moiety and the hexyl group, and the methyl side-chain end-groups are found between 2.4–1.19 ppm (multiplets) and at 0.79 ppm (singlet), respectively [42]. The extent of regioregularity of P3HT evaluated from the peak at 2.48 ppm was found to be 84 %. Average molecular weight, *M_n_*, and dispersity, *PDI* were estimated from GPC/SEC technique. *M_n_* was found to be 16,239 g/mol and the average molecular weight, *M_w_* 30,315 g/mol. The dispersity, *PDI* = *M_w_/M_n_* is calculated to be 1.87. Further structural investigation was carried out using FTIR. G1PPT-co-P3HT vibrational frequencies spectrum analyzed with reference to 3-hexylthiophene (3-HT) and G1PPT, is found in Figure 2. The star copolymer spectrum is characterized by many bands associated with the different molecular vibrations within the material which are found at 2954-2849, 1731, 1638, 1473, 1262, 802, and 692 cm^−1^. Bands in the spectral region 2954–2854 cm^−1^ are characteristics of aliphatic −C−H stretching signals of the hexyl group attached to the thiophene rings [43]. At 1731 and 1638 cm^−1^ are the bands resulting from the molecular vibrations of C=N−H and C=C, respectively; the same vibrations were already observed in G1PPT at 1673 and 1632 cm^1^ with high intensity [44]. The C−H bending bands already present in G1PPT and 3-HT at 1432 and 1462 cm^−1^, respectively, are found at 1473 cm^−1^ in G1PPT-co-P3HT. At 802 cm^−1^, the C−S stretching is within the thiophene rings that was also observed in 3-HT and G1PPT [45]. Finally, the lower vibrational band at 692 cm^−1^ in G1PPT-co-P3HT, already present in G1PPT at 703 cm^−1^, is as a result of the alkyl groups -C-H bending vibrations of the proton at α-position with respect to the non-alkylated thiophene ring and next to the imine bond [36,46]. Molecular vibrational frequencies in G1PPT-co-P3HT, 3-HT and G1PPT are summarized in Table 1.

#### 3.1.2. Morphological and Particle Size Investigation

**Figure 3:**
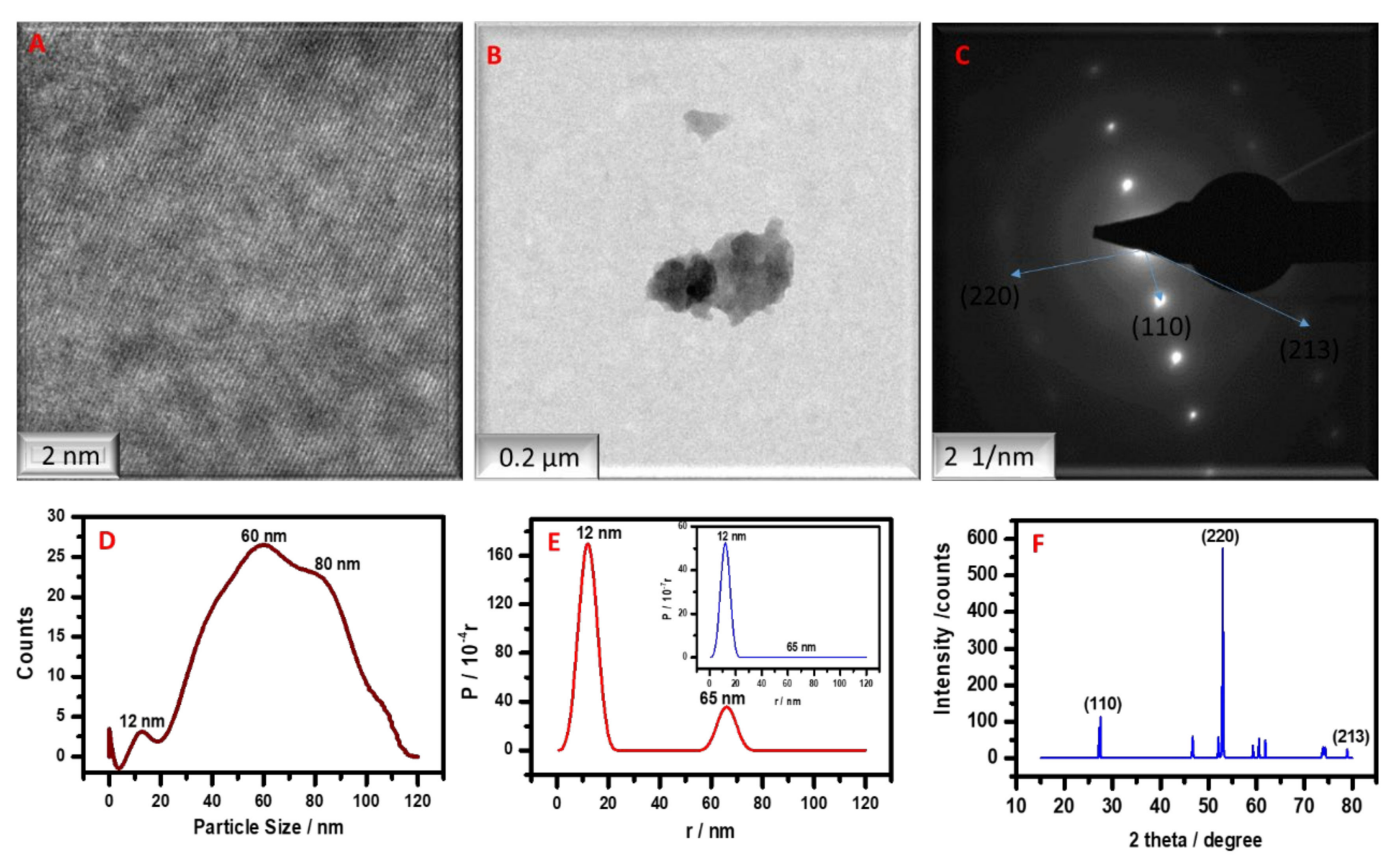
HRSEM (**A**,**B**) and SAED (**C**) images of G1PPT-co-P3HT, SAXS PDDF (**D**), size distribution function by volume and by number(inset) (**E**) and XRD pattern (**F**) of G1PPT-co-P3HT [1].

HRTEM was used as the technique of choice for the morphological investigation of the star copolymer. The results depicted on the images in Figure 3A,B represent the morphology of G1PPT-co-P3HT thin film casted from chloroform. Despite 84% regioregularity of the star copolymer as demonstrated by ^1^H NMR spectroscopy, G1PPT-co-P3HT HRTEM images show various regions with well-defined lattice fringes. The crystalline regions were confirmed by the SAED (Selected Area Electron Diffraction) image (Figure 3C) in which well-patterned crystal lattice arrays were observed; therefore, confirming our hypothesis from NMR data that the P3HT component in the star copolymer is composed of a higher ratio of H-T rr-P3HT compared to H-H and/or T-T ra-P3HT. The d-spacing values were evaluated using the image-processing software ImageJ and suggests that the macromolecule presents a body-centered cubic (BCC) structure. G1PPT was polycrystalline (Figure S4, Supplementary Materials) and 3-HT was quite amorphous with coarse nature (Figure S5, Supplementary Materials), it is therefore believed that copolymerization of 3-HT to the functionalized dendrimer further improves the molecular ordering in the star copolymer. Since crystallinity is a very important and critical factor that affects the properties of optoelectronic films, the achieved degree of crystallinity could assist in attaining good organic photovoltaic performances [47]. The objective of attaining a certain degree of crystallinity is the reason why the authors decided to move away from attaching PEDOT to G1PPT, as its star copolymer G1PPT-co-PEDOT remained amorphous as reported in a previous paper [36].

Small-angle X-ray scattering (SAXS) was used to obtain the particle size distribution of G1PPT-co-P3HT. The free-model pair distance distribution function of the star copolymer depicted in Figure 3D shows that G1PPT-co-P3HT is mostly in aggregated form, with a maximum particle size of ~80 nm. The pair-distance distribution functions of the nanoparticles in volume-weighted particle size distributions are plotted in Figure 3E, with the number-weighted particle size distribution as inset. In general, nanoparticles in the ranges of 2–23 nm and 55–76 nm are described by scattering cross-sections, with a larger portion of the nanoparticles at 12 nm for the number-weighted distribution. The volume-weighted particle size distribution shows the population of particles seen by their volume. The predominance of smaller particles of 12 nm contradicts the common knowledge that larger particles are ‘more seen’ than the smaller particles [48].

X-ray diffractogram of G1PPT-co-P3HT, Figure 3F presents various Bragg’s peaks with the most intense at 2ϴ = 52.997°. All the peaks are attributed to the presence of various crystalline regions with different grains sizes. The miller indices at 27.18, 52.997, and 78.88° correspond to (110), (220), and (213), respectively, and correlate to the values of a BCC Bravais lattice, confirming the hypothesis from SAED results. The average crystallite size was calculated from the (220) Bragg’s peak, using Scherrer’s formula [49] below:

D=Kλ/βcosϴ

where *D* is the mean crystallite size, *K* is the shape factor constant (0.89 rad), *λ* is the wavelength of the X-rays, *β* is the full width at half maximum (FWHM) of the (220) Bragg’s peak, and *ϴ* is the angle of reflection of the (220) peak. The calculated grain size was 12.6 nm which agrees with the results obtained from SAXS analysis.

#### 3.1.3. Optical and Photo-Physical Characterization 

**Figure 4:**
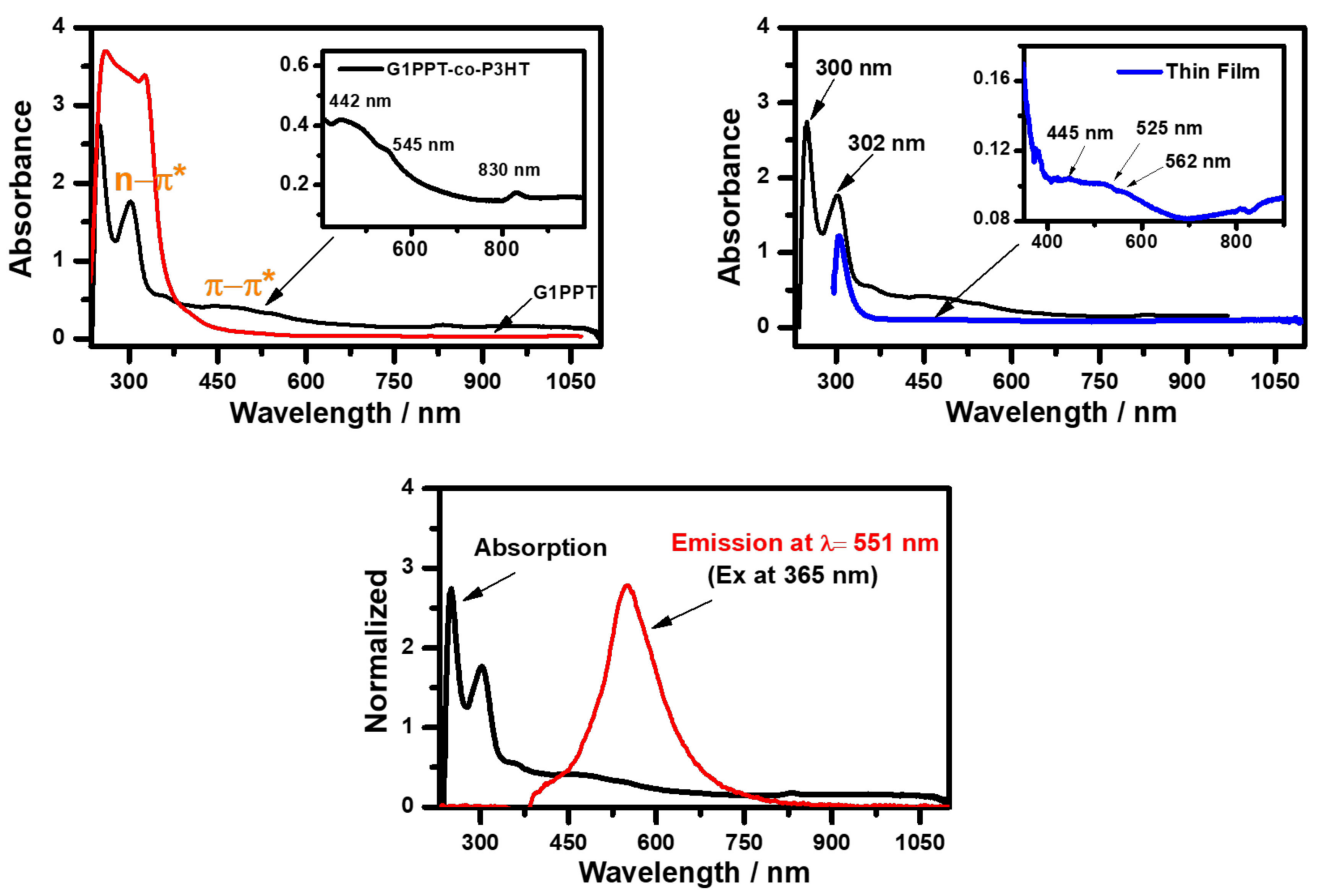
UV-Vis absorption spectra of G1PPT-co-P3HT and G1PPT in chloroform solution (**top left**), as thin film (**top right**) and photoluminescence spectrum (**bottom**) [1].

Absorption properties of 725.5 µM G1PPT-co-P3HT investigated in solution from chloroform as well as on spin-coated thin films are found in Figure 4. The optical band gap energy, $E_{g}^{opt}$ value calculated to be 1.43 eV was obtained by determining the onset at the higher wavelength region using the equation 1242λonset. The star copolymer, G1PPT-co-P3HT displayed combined absorption properties of G1PPT and P3HT with an extinction coefficient of ca. 4441 mol^−1^.cm^−1^. This was characterized by two strong absorbance bands in the ultraviolet (UV) region with absorption maximum at 251 nm; and two new bands in the visible region. The two intense absorption peaks at higher frequency are due to the n-π* electronic transitions in the C=C within the thiophene moiety [45], whereas the broad band between 423 nm and 676 nm, with maximum absorption at ca. 442 nm and a shoulder at 545 nm is as a result of the π-π* electronic transition in the conjugated polymer backbone [50]. We suggest that this band could also be due to intramolecular charge transfer (ICT) caused by the delocalization of the electrons from the star copolymer core through to the extended conjugated P3HT dendrons [51]. The presence of the shoulder results from the co-existence of various phases of different conjugation length in the star copolymer [52]. At higher wavelength, in the NIR region at 830 nm, another peak is observed with an absorption onset at 870 nm from which the energy band gap was calculated. This peak is suggested to be due to the existence of some doped states of P3HT within the macromolecular structure of the star-copolymer [53,54]. On the other hand, solid-state G1PPT-co-P3HT demonstrated continuous absorption over the whole spectral range from 300 to 1100 nm with the lowest absorption intensity recorded around 700 nm. All absorption peaks were red-shifted compared to the peaks of G1PPT-co-P3HT in solution, with the broad band being wider, owing to strong inter-chain interactions and π-π stacking in the solid state which are beneficial for charge transport [55]. Additionally, another shoulder at 525 nm is noticeable which suggests that in the solid state, there is occurrence of a new phase, probably as a result of a new stacking orientation. The shoulder at 565 nm can be assigned to the vibronic structure with a 0-0 transition, whereas the vibronic side-band is at 525 nm [52]. It has been reported that poly(3-alkylated thiophene)s, P3ATs which have a higher tendency to orient in a coplanar fashion with extended π-conjugation along its backbone, can only be justified by their completely regioregular H-T couplings and shows the absorption maximum is at 560 nm. P3ATs with lower percentage of H-T couplings form thin films with broad absorption maxima around 480 nm, in the solid state [50]. This calls for our attention to note that, even though the optical band gap energy of G1PPT-co-P3HT is lower than reported P3HT band gap of 1.9 eV, the peaks of P3HT within the star copolymer are all blue-shifted with respect to reported pristine P3HT, whether regioregular or regiorandom. This shows that the molecular bulkiness of the star copolymer coupled to the presence of H-H and/or T-T results in steric hindrance and twisted orientation. In addition, similar to P3HT that has demonstrated solvatochromic processes from yellow to dark purple, G1PPT-co-P3HT also exhibits solvatochromism with a change of color from green to black strongly associated with the presence of more ordered species, i.e., alkyl chains, having stronger π-electron orbital overlap between adjacent thiophene rings [56]. The preference for P3HT over PEDOT as the dendritic units in star copolymers is therefore justified. Indeed, as previously reported, the absorption range of G1PPT-co-P3HT counterpart, namely, G1PPT-co-PEDOT was only up to about 700 nm with an optical band gap of around 1.8 eV [36].

The photoluminescence spectrum of G1PPT-co-P3HT when excited at a wavelength of 365 nm is shown in Figure 4, bottom. Photoluminescence quenching studies were carried out to examine the exciton behavior. While the star copolymer demonstrated many absorption bands in the UV-Vis and the NIR region during spectroscopic studies, only one intense photoluminescence peak was observed when the material was exposed to monochromatic light-emitting diode light source of wavelength equals to 365 nm using an Ocean optics device. Upon excitation, the electrons were found to relapse to ground state via light emission at 551 nm, causing a Stokes’ shift of 300 nm. Such large Stokes’ shift already predicts the formation of aggregates in solid state [57,58]. Emission at higher wavelength region results from intramolecular charge transfer (ICT) caused by effective π-conjugation [59].

#### 3.1.4. Electrochemical Characterization

**Scheme 3:**
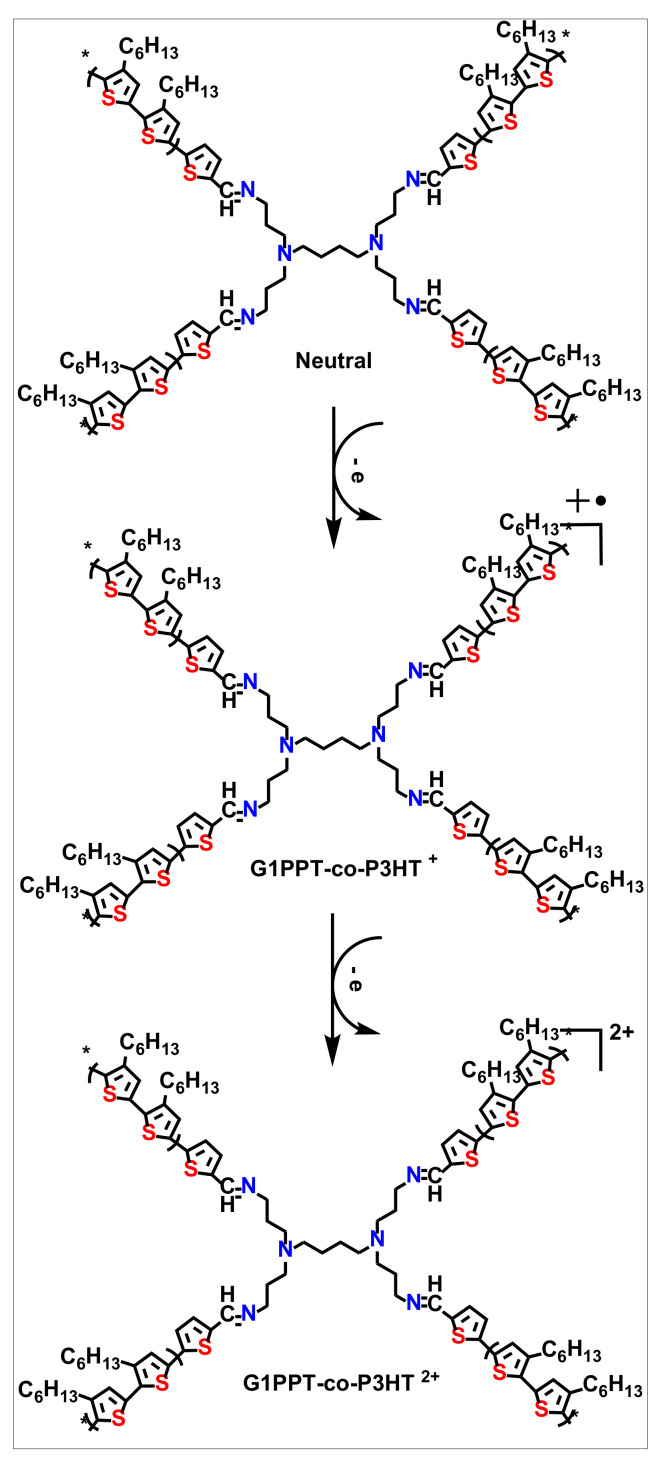
Two-step redox process of G1PPT-co-P3HT|Au electrode in in 0.1 Bu_4_NPF_6_ in acetonitrile [1].

**Figure 5:**
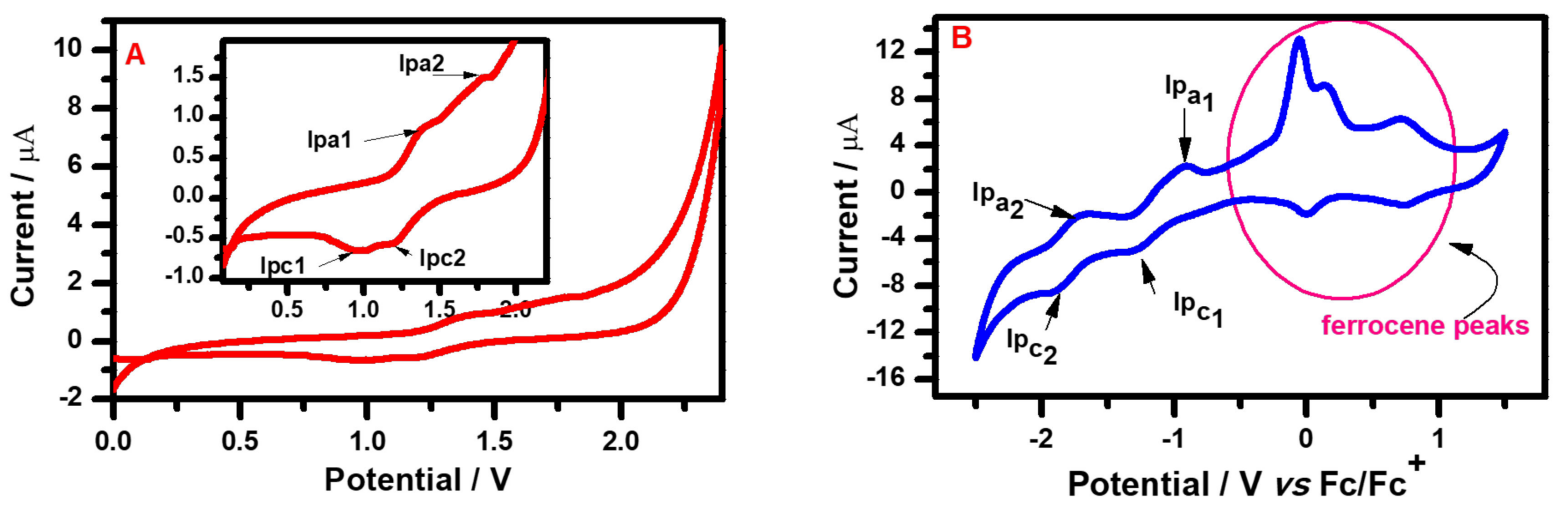
Cyclic voltammogram of G1PPT-co-P3HT in acetonitrile containing 0.1 M Bu_4_NPF_6_ at scan rate 50 mV/s using Ag/AgCl reference electrode (**A**) and versus Ag/Ag+ reference electrode in ferrocene (**B**) [1].

G1PPT-co-P3HT electrochemical behavior was studied using cyclic voltammetry (CV) and electrochemical impedance spectroscopy (EIS). Typical voltammetric behavior of G1PPT-co-P3HT, swept in a potential window between 0 and 2.5 V at a scan rate of 50 mV/s, is depicted in Figure 5A and characterized by two quasi-reversible redox couples, similar to our previously reported G1PPT-co-PEDOT [36]. The two oxidation peaks, ip_a1_ and ip_a2_ are found at 1.38 and 1.73 V, respectively, while the two reduction peaks, ip_c1_ and ip_c2_, are found at 0.97 and 1.21 V, respectively. Anodic peaks of P3HT have been reported to be found at a less positive, below 1 V. This shows that copolymerization to G1PPT has resulted in the oxidation processes to occur at higher positive potentials. Reports also demonstrated that one, two, and sometimes three anodic peaks could be detected in the cyclic voltammograms of poly(alkylatedthiophene)s, PATs depending on the extent of regioregularity of the conjugated chain [60]. While one anodic peak has been assigned to a regiorandom PAT with regioregularity below 60%, the appearance of two peaks, usually exhibited by PATs with regioregularity between 70–90%, can be justified using two different approaches. The first approach suggests the existence of a two-step process: (1) the oxidation of the neutral polymer to radical cations and, (2) the oxidation of the radical cations to dications. In the second approach, the occurrence of two consecutive redox peaks is as a result of the co-existence of regions of different conjugation lengths and/or various molecular orderings (morphology), i.e., crystalline and amorphous [52]. While the oxidation located at the most positive potential is ascribed to oxidation of amorphous, short conjugated chains, the peak at lower anodic potential of the cyclic voltammogram is attributed to electron loss within the polymer zones with the longest average conjugation length. On the other hand, a third anodic peak in PATs could only arise from high regioregularity above 95%. This voltammetry information therefore confirms the extent of regioregularity, between 80–90%, of the P3HT component within the star copolymer. The mechanism of electrons loss in the star copolymer as per the first approach is depicted in Scheme 3.

Further cyclic voltammetric analyses of G1PPT-co-P3HT on gold-modified electrode versus Ag/Ag^+^ in the presence of ferrocene (Figure 5B) was conducted to determine its frontier orbital energies: highest occupied molecular orbital (HOMO) and lowest unoccupied molecular orbital (LUMO), and electrochemical energy band gap. Two main groups of peak sets were observed. Two redox couples, *ip_a_*_1_ and *ip_c_*_1_, and *ip_a_*_2_ and *ip_c_*_2_ attributed to the redox processes undergone by G1PPT-co-P3HT can be observed in the negative potential region of the spectrum, while the three oxidation and two reduction peaks in the positive potential region of the spectrum are suggested to be due to ferrocene. According to Koopman’s theorem, frontier orbital energies can be used to approximate many OPVs properties that include ionization potential energies, *IP*, electron affinities *EA*, electronic/electrochemical band gap, $E_{g}^{ec},$ open circuit voltage, *V_OC_*, and the driving force for charge separation. The most common approximation involving frontier orbitals is to assume that *E_HOMO_* is equivalent to the ionization potential energy, *E_IP_* and *E_LUMO_* is equivalent to the electron affinity energy, *E_A_* [61]. *HOMO* and *LUMO* energy levels were estimated from their respective first oxidation and reduction onset potentials, *E_onset_*, on the reference of ferrocene, Fc/Fc^+^ energy level (4.8 eV below the vacuum level). The calculations were made using Bredas et al., [61] equations:

(1) $$E_{A},\;LUMO=-e\left(E_{onset}^{red}-E_{ferr}\right)V-4.8\;\mathrm{eV}$$

(2) $$E_{IP},\;HOMO=-e\left(E_{onset}^{ox}-E_{ferr}\right)V-4.8\;\mathrm{eV}$$

(3) $$E_{g}^{ec}=|E_{IP}-E_{A}|$$

where, *E_ferr_* = −0.03 V is the value for ferrocene vs the Ag/Ag+ electrode. 

First oxidation and reduction onsets were found to be −0.63 and −2.2 V, respectively. From which *E*_IP_ and *E*_A_ were calculated to be 5.53 and 3.6 eV, respectively, giving rise to an electrochemical band gap $E_{g}^{ec}$ = 1.73 eV, slightly higher than the optical band gap, $E_{g}^{opt}$ = 1.43 eV calculated from the absorption wavelength onset. The difference observed between $E_{g}^{ec}\;\mathrm{and}\;E_{g}^{opt}$ arises from significant interface dipoles not taken into account during the calculations that alter the frontier energies [52]. The HOMO energy value of G1PPT-co-P3HT at −5.53 eV, below the air-oxidation threshold of 5.3 eV, proves the air-stability of the star copolymer which is one of the attributes of an ideal polymeric material for OPVs [62]. In addition, such relatively low HOMO of the star copolymer may lead to a high open circuit potential (*V_OC_*) value in an organic photovoltaic cell [63]. 

**Figure 6:**
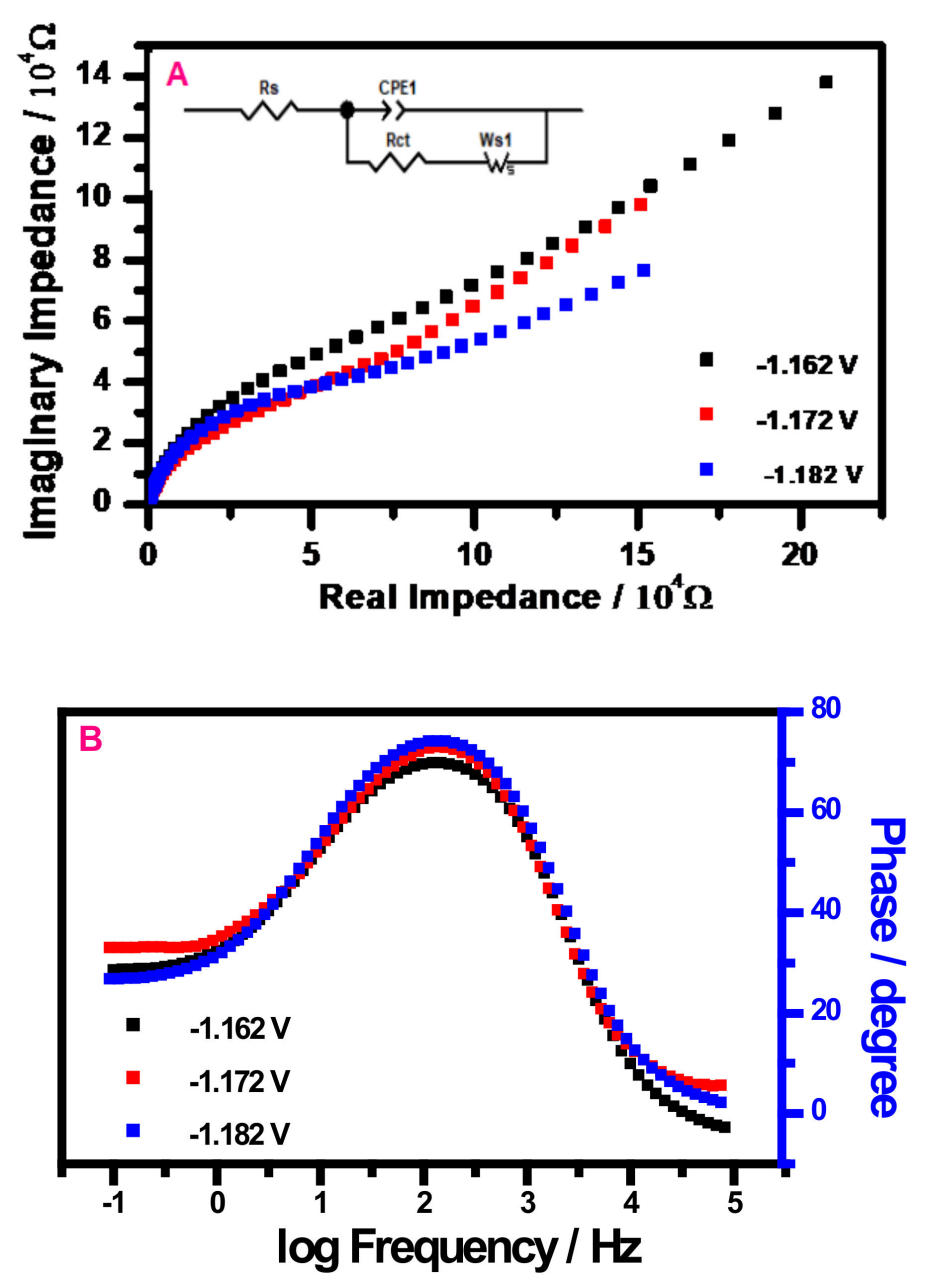
Nyquist plot (**A**) and Bode plot (**B**) of G1PPT-co-P3HT in 0.1 M Bu_4_NPF_6_ in acetonitrile at 1.182 V [1].

Electrochemical impedance spectroscopy (EIS) measurements were used to investigate the electrical resistance and diffusive processes occurring at the electrochemical interface of the electrode. Figure 6 shows the Nyquist plot (A) and Bode plot (B) of G1PPT-co-P3HT and the equivalent circuit model (the inset in Figure 6A) used for fitting the EIS data. The star copolymer was deposited on a working gold electrode disc in 0.1M Bu_4_NPF_6_ (acetonitrile) at a scan rate of 50 mV/s using a three-electrode system with three different applied potentials of −1.162, −1.172, and −1.182 V. The Nyquist plots consist of two distinct parts: a small semi-circle in the high frequency region which describes the electron transfer-limiting processes and an inclined line in the medium to low frequency region which provides information about the diffusion-controlled processes [64]. The intercept at the high frequency zone along the *Z*’ axis corresponds to the Ohmic/solution resistance (*R*_s_), while the diameter of the semicircle along the *Z*’ axis relates to the charge transfer resistance (*R_ct_*), which was obtained after fitting the data using the equivalent circuit. The inclined line is attributed to the diffusion of tetrabutylammonium ions in the bulk of the extended conjugation known as the Warburg diffusion (*Z_W_*). The resulting parameters obtained from fitting simulation are recorded in Table 2. The *R_c_*_t_ decreased with increasing applied potential, i.e., the lowest *R_ct_* was exhibited by the highest applied formal potential at −1.182 V. This indicates a faster charge transfer kinetic process comparatively to the other two applied potentials. For a pure capacitor, a vertical line should be exhibited at low frequency, i.e., phase angle at 90°. Deviation from the vertical line is attributed to the inner diffusion resistance for electrolyte ions that reduces phase angle degree, and which is usually strongly dependent on the applied formal potential [65]. Microscopic roughness of the electrode surface, and slow adsorption of ions and surface inhomogeneity also make it practically impossible for the barrier film (double-layer) to exhibit the theoretically expected phase angle value of 90° [66]. G1PPT-co-P3HT displayed increasing phase angle degrees with increasing applied potential in his Bode plot, which is in agreement with results obtained from the Nyquist plot. Obtained phase angles above 70 degree demonstrate the relatively good semiconducting properties of the star copolymer. The constant phase element (*CPE*) which describes the double-layer capacitance of the electrode-solution interface, a parallel parameter to *R_ct_* and *Z*_W,_ is found not to be a function of the applied potential. 

### 3.2. Bulk Heterojunction Blends and Photovoltaic Performances

#### 3.2.1. Optical and Photo-Physical Investigation of G1PPT-co-P3HT:PDI-co-Carbazole Blends

**Figure 7:**
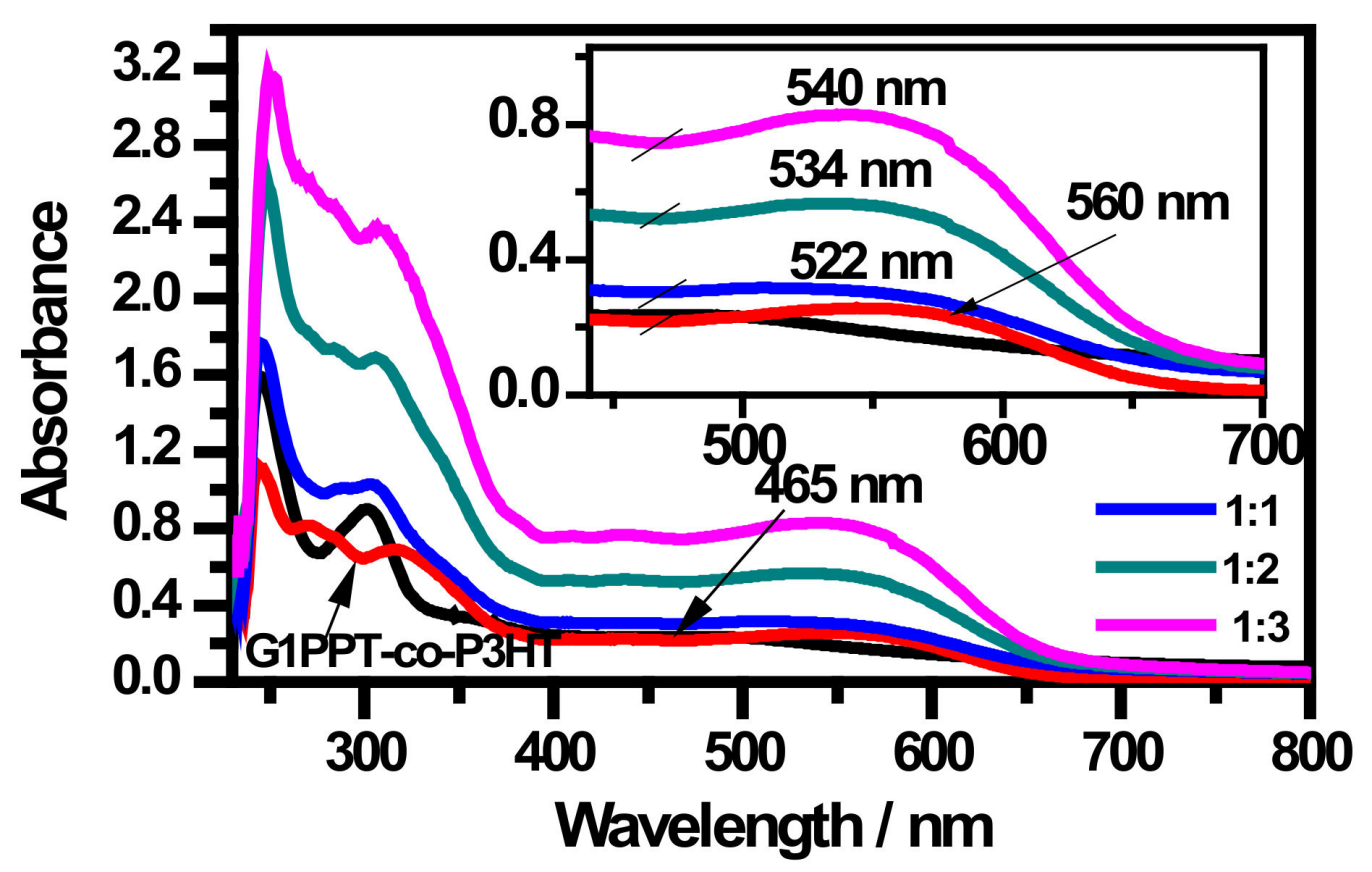
UV-Vis absorption of G1PPT-co-P3HT/PDI-co-Carbazole at different ratio [1].

Optical absorbance properties of G1PPT-co-P3HT:PDI-co-Carbazole bulk heterojunction blends were studied in chloroform within the UV-Vis-NIR spectral region as depicted in Figure 7. The blends were prepared in the ratio 1:1, 1:2, and 1:3 and investigated with reference to pristine donor G1PPT-co-P3HT (donor) and pristine acceptor PDI-co-Carbazole (acceptor). The blends displayed combined optical behavior of both pristine polymers, with strong absorption in the UV-Vis spectral region and onset absorption around 675 nm. It was interesting to note for all BHJ blends, the absorption maxima, *λ_max_* for the broad band between 470–680 nm corresponding to the intermolecular charge transfer (ICT) [51] shifted to shorter wavelength (blue shift) with respect the pristine PDI-co-Carbazole and to a longer wavelength with respect to pristine G1PPT-co-P3HT. Indeed, as the amount of PDI-co-Carbazole with respect to G1PPT-co-P3HT was increased in the ratios 1:1, 1:2, and 1:3, the red shift of the blends with respect to the donor were 57, 69, and 75 nm in 1:1, 1:2, and 1:3, respectively. This therefore suggests that there is an effective electron transfer between the electron-donating polymer and the electron-accepting PDI-co-Carbazole. On the other hand, the blue shift of the blends with respect to pristine PDI-co-Carbazole with the 1:1 ratio having 38 nm blue shift, suggests that addition of the later to the pristine G1PPT-co-P3HT results in the deplanarization of PDI-co-Carbazole [67], but further addition of the electron-accepting polymer might favor strong co-facial π-π interactions that create some molecular ordering [15].

**Figure 8:**
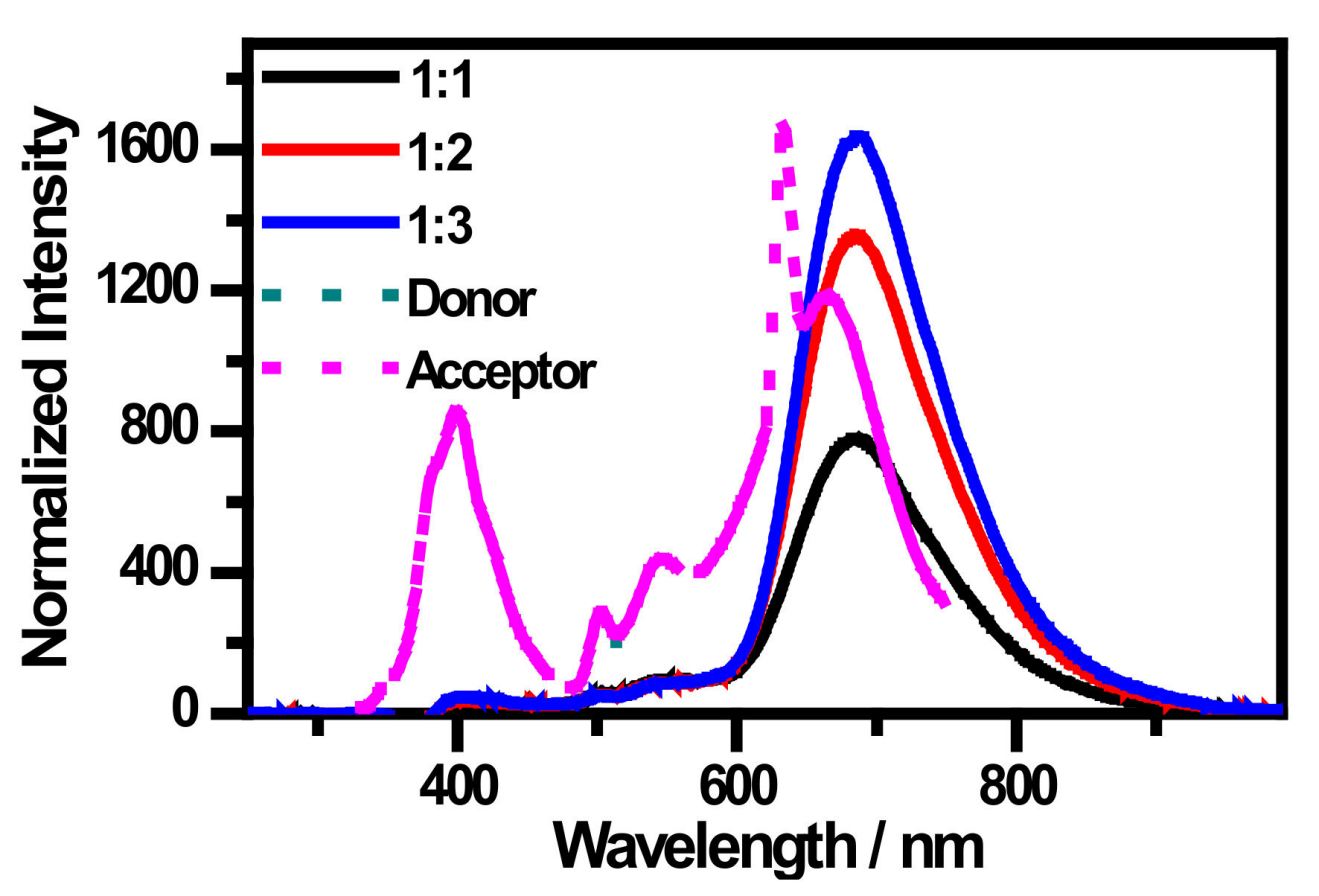
Emission spectra of G1PPT-co-P3HT, PDI-co-Carbazole and their blends [1].

**Figure 9:**
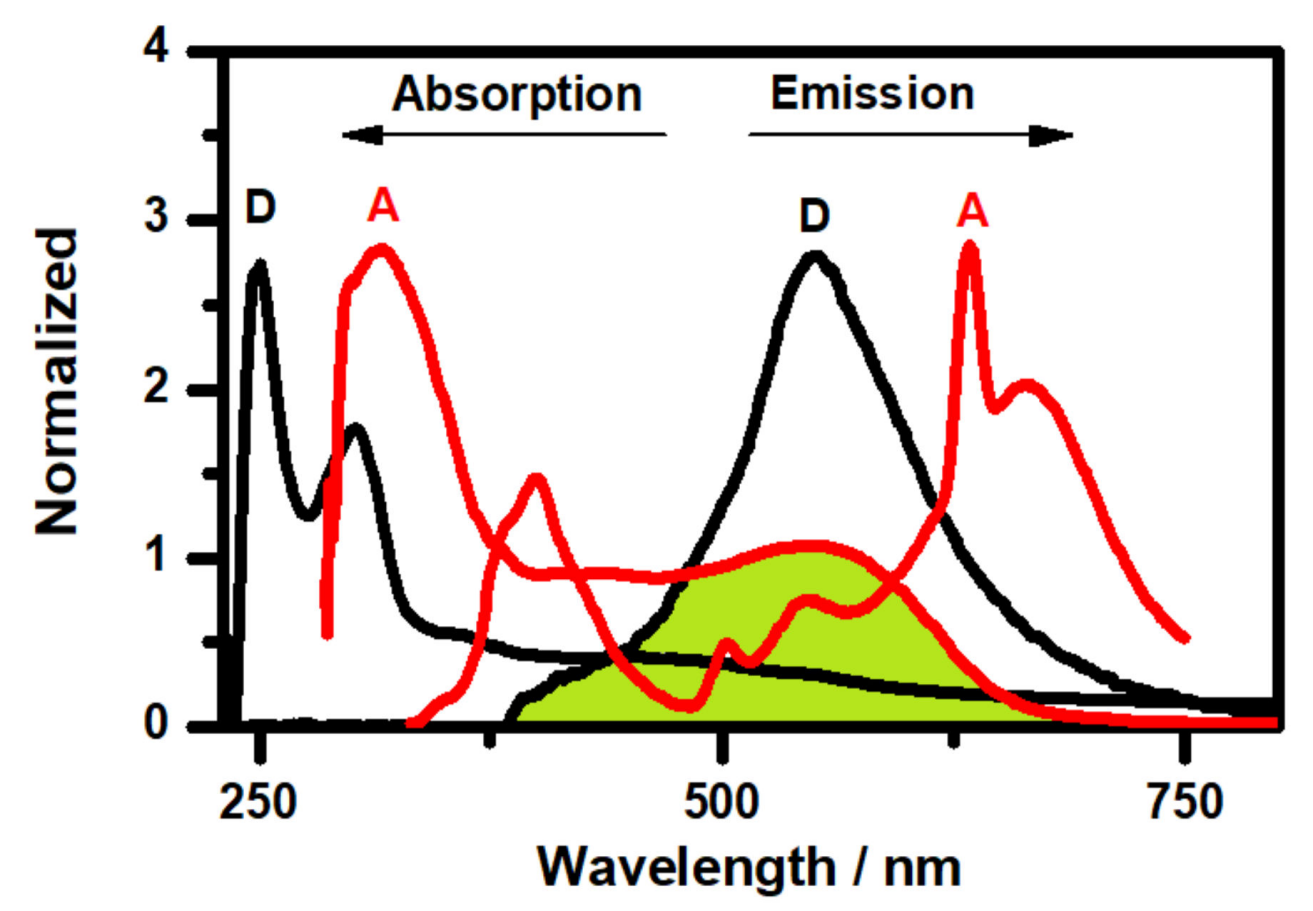
Absorption and emission spectra of G1PPT-co-P3HT (D) and PDI-co-Carbazole (green area represents the spectral overlap of donor emission and acceptor absorption characterizing the Förster resonance energy transfer) [1].

G1PPT-co-P3HT/PDI-co-Carbazole different bulk heterojunction blended photo-physical properties were also investigated using photoluminescence the spectroscopic technique. The nanocomposite fluorescence properties were studied. Fluorescence of pristine G1PPT-co-P3HT and PDI-co-Carbazole are presented in Figure 8 Upon excitation of D-A blends, total quenching of the donor is observed, characterized by the complete disappearance of the G1PPT-co-P3HT fluorescence emission peak initially exhibited by the pristine material at 551 nm, an indication that there is essentially no radiative exciton decay in the blend [68]. This can be as a result of the photo-induced electron transfer between the electron-donating G1PPT-co-P3HT and the electron-accepting, PDI-co-Carbazole which is the desired process in photovoltaic devices. Additionally, while a red shift (20 nm) in the emission peak of the blends is observed from 665 nm in pristine PDI-co-Carbazole to 685 nm in the blends, an increase in peak intensity with increasing content of PDI-co-carbazole can also be noted. The total disappearance of the donor fluorescence peak coupled to the increase in acceptor intensity peaks suggest that there is also a Fluorescence or Förster resonance energy transfer (FRET) from the donor to the acceptor [69]. This is further confirmed by the emission spectrum of the donor overlapping the acceptor absorption spectrum [70] as shown in Figure 9. We also suspect that this energy transfer is followed by non-radiative recombination [69] considering the increase in acceptor peak intensity as mentioned above.

#### 3.2.2. I-V Curve Characteristics and Morphology–Device Performance Relationship: Preliminary Studies

**Figure 10:**
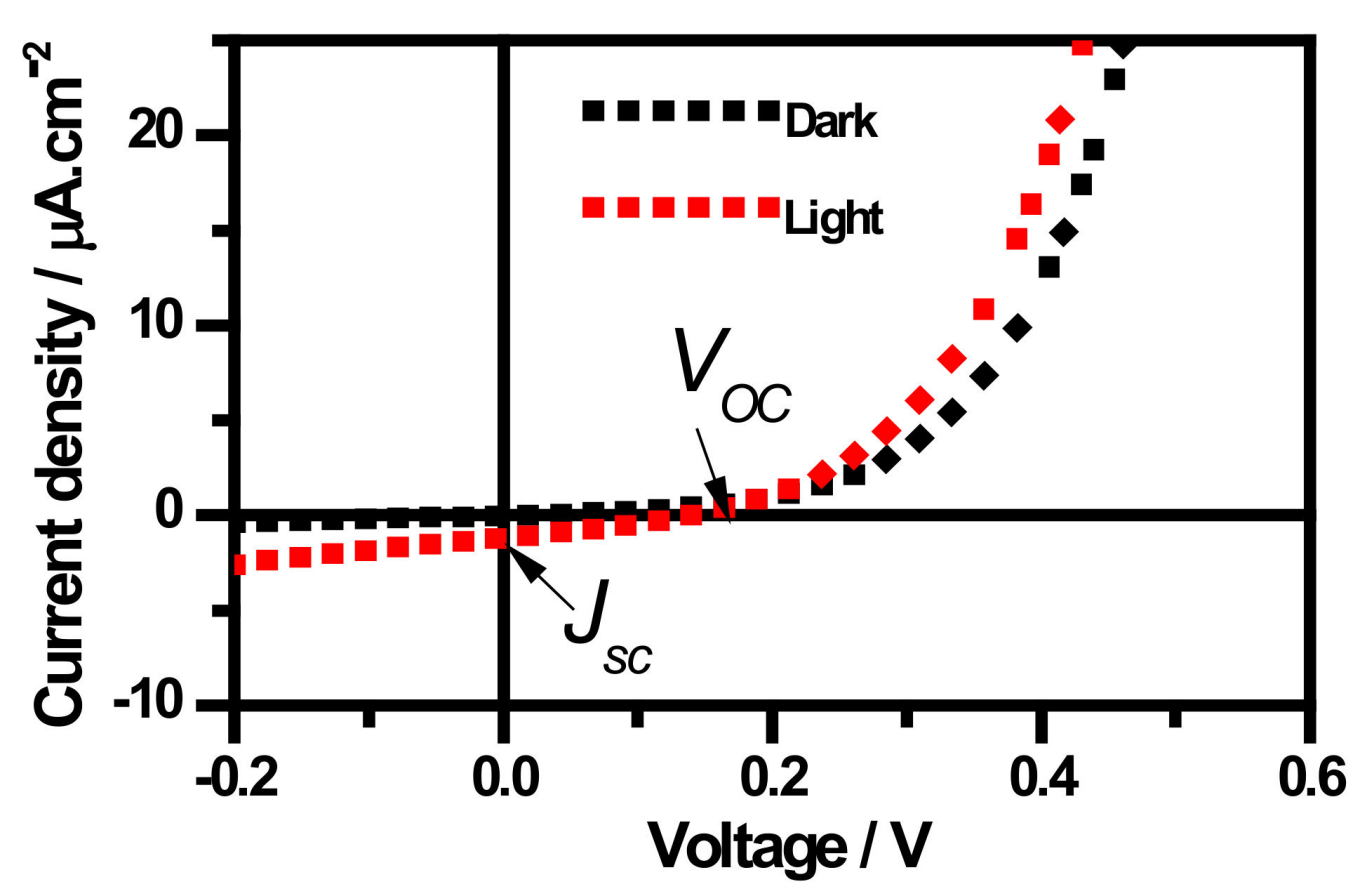
Typical I-V curve characteristic of prepared devices [1].

Preliminary investigation of the application of these BHJ layers into solar cells was conducted. Typical I–V curve characteristics of the BHJ devices are depicted in Figure 10. The parameters of the PSCs are summarized in Table 3. All devices exhibited performances below 1% except for P3HT:PC_61_BM (used as a reference) that achieved a power conversion efficiency of 1.5% with *V_OC_* = 510 mV, short-circuit current density, *J_SC_* = 7.7 mA/cm^2^, fill factor, *FF* = 0.38. G1PPT-co-P3HT:PDI-co-Carbazole (2:1) exhibited the lowest characteristics with *V_OC_* = 50 mV, *J_SC_* = 2.65 µA/cm^2^, fill factor, *FF* = 0.11 which resulted in a power conversion far below 1%. Parameters of the device based on G1PPT-co-P3HT with the fullerene derivative PC_61_BM in a 1:2 ratio were slightly higher than in the case of the all-polymer device. However, the efficiency is still found to be far below 1%. It is commonly known that an ‘ideal’ exciton-splitting energy, the difference between the LUMO of electron-donating material and LUMO of electron-accepting material, 0.3 eV, is required to ensure proper charge-carrier separation. Although the exciton-splitting energy of G1PPT-co- P3HT:PDI-co-Carbazole (2:1) is calculated to be lower ((−3.6–(−3.87) eV) = 0.27 eV) compared to that of the device fabricated with G1PPT-co-P3HT: PC_61_BM (1:2) i.e., 0.5 eV where, PC_61_BM LUMO energy level is −4.1 eV [71], the obtained efficiency of the latter is believed to be slightly higher due to the rigidity of PDI-co-Carbazole polymeric molecule that hinders continuous charge-carrier diffusion. Indeed, the presence of straight dodecyl chains in the electron-accepting polymer hinders the flexibility of the molecule, which in turn has an impact on the extent of interchain interaction between the electron-donating and accepting polymers and on the overall device performance [72]. It is therefore most likely that recombination of the separated charges (holes and electrons) immediately after their excitation through light absorption occurred. This was already predicted from fluorescence blend results. Indeed, the spectral overlap of the donor, G1PPT-co-P3HT emission spectrum, and the acceptor, PDI-co-carbazole absorption spectrum, was a sign of an energy transfer. Mostly, the increase in fluorescence intensity peak of the acceptor suggested that non-radiative recombination occurs [73]. Another device based on PDI-co-Carbazole with P3HT was also fabricated to investigate if commercially available regioregular, 94% rr-P3HT could allow better performance. Indeed, switching from G1PPT-co-P3HT to rr-P3HT increased the *V_OC_* to 180 mV, the *J_SC_* to 62 µA/cm^2^ and *FF* = 0.26. The efficiency was thus increased by one order of magnitude.

Various studies have demonstrated that organic photovoltaic device performances are strongly dependent on the BHJ blend morphology [74,75]. It is therefore imperative to understand the relation between them. In order to achieve good charge-carrier generation, the interface between the donor and the acceptor should be large enough and an efficient charge extraction should be allowed by the networks in the blend of donor and acceptor polymers [76]. In addition, the capacity of both components to exhibit crystalline ordering on length scales of several nanometers could be beneficial for charge-carrier transport and device efficiency. Thin films morphologies of the fabricated devices were investigated by SEM microscopy (Figures S7–S10 in Supplementary Materials). The images showed that in most devices, the blends were characterized by defects that act as charge trappings inhibiting continuous flow of the charges through to the respective electrodes. Precise morphology parameters, including the van der Waals crystal packing of the donor and acceptor polymers; and the formation of nanoscale domains of the two phases, are strongly dependent on the donor and acceptor blend ratio, the solvents used, processing conditions, and finally post-production treatment [77]. The use of chloroform as the casting solvent could therefore have played a detrimental effect on the morphology of the devices as the fast evaporation of the solvent resulted in material clustering. In addition, the tendency of both G1PPT-co-P3HT and PDI-co-Carbazole to self-aggregate during film deposition can also account for the isolation/trapping of the charges within the macromolecule lattices [78,79]. G1PPT-co-P3HT:PDI-co-Carbazole (2:1) device FF of 0.11, smaller than those of other devices may be due to short-conjugated length and amorphous structure within G1PPT-co-P3HT. Usually, low FFs (below 0.4) of solution processed OPVs are also associated with large series resistance and small shunt resistance [80]. In OPVs, the device performance is strongly dependent on free charge-carrier generation, transport in the active layer, and collection at corresponding electrodes. The I–V characteristics of most devices exhibit strong electric field dependence associated with recombination processes. This is probably induced by the energetically close positions of G1PPT-co-P3HT and PDI-co-Carbazole [80], thereby reducing the selectivity of the negative contact to separate holes and electrons. Such behaviors are good intrinsic characteristics of organic light-emitting diodes.

## 4. Conclusions

For the first time, oxidative copolymerization of functionalized dendrimer, G1PPT to poly(3-hexylthiophene) in the presence of FeCl_3_ in chloroform was successfully achieved as confirmed by 1H NMR and FTIR spectroscopic analyses of the prepared dendritic star copolymer, G1PPT- co-P3HT. The analyses demonstrated several characteristic chemical shifts and vibrational bands associated with the features of poly(3-hexylthiophene) and the functionalized dendrimer. Cyclic voltammetric, microscopic, and optical spectral data were used to confirm the degree of regioregularity and type of morphologies, while prepared with high molecular weight, *M*_w_ 30,315 g/mol, the star copolymer exhibited an optical band gap energy as low as 1.43 eV, which falls within the optical band gap window of an ideal donating material in OPVs. The two shoulders, at 525 and 567 nm observed in G1PPT-co-P3HT solid-state optical thin film and associated with vibronic side-band and vibronic 0-0 structure, respectively, attested to the existence of different morphologies within the material. This was further confirmed when the G1PPT-co-P3HT-modified Au electrode exhibited two oxidation peaks in the positive potential region during voltammetric studies. These two anodic peaks ascertained the existence of regioregularity between 80–90% caused by amorphous polymer zones of shortly conjugated chains and more ordered regions with longer average conjugation length. The calculated HOMO frontier orbital of −5.53 eV, which confers an air-stability property, also suggested that this dendritic material could exhibit good open-circuit voltage, *V_OC_*, a key parameter for optimal OPVs performance. Further electrochemical investigation through EIS analysis, proved that G1PPT-co-P3HT has good semiconducting properties with phase angle above 70°. The semi-crystalline nature of the prepared dendritic star copolymer coupled to the exhibited electronic properties therefore qualified G1PPT-co-P3HT as a good electron-donating candidate for optoelectronic devices. Preliminary studies of I-V characteristics of fabricated devices based on G1PPT-co-P3HT and PDI-co-Carbazole with observable *V_OC_* suggest that if the morphologies and ratio are improved, and they could exhibit outstanding PCE; it also shows the applicability of these bulk heterojunction layers for organic light-emitting diodes.

## Figures and Tables

**Table 1:** **Molecular vibrational frequencies in G1PPT-co-P3HT, 3-HT, and G1PPT** [1].

Origin	Group Frequency (cm^−1^)			Vibrational Mode
	G1PPT-co-P3HT	3-HT	G1PPT	
C−H	2954–2849	2967–2855	2961–2801	−C-H stretching
	1473	1462	1432	−C-H bending
	692	-	703	−C-H bending, (α-position to the ring)
C=C	1638	1632, 1538 (weak)	1632	−C=C stretching
C=N	1731	-	1673	−C=N-H stretching

**Table 2:** Electrochemical parameters of G1PPT-co-P3HT obtained from EIS measurements [1].

Eʹ/V.	*R_s_*/Ω	*R_ct_*/kΩ	*CPE*/µF	Phase Angle/Degree
−1.162	289.7	42.67	0.92	70.1
−1.172	290.4	34.29	0.91	73.3
−1. 182	291.1	25.66	0.92	75.4

**Table 3:** Summary of photovoltaic device performances. Device structure: ITO/PEDOT:PSS/Active layer/Al [1].

* Active Layer *	*V_OC_* *(mV)*	*J_SC_* *(µA/cm^2^)*	* FF *	*PCE/* *(1 × 10^−3^%)*
* P3HT:PDI-co-Carbazole (2: 1) *	* 180 *	* 62 *	* 0.26 *	* 3.0 × 10^1^*
* G1PPT-co-P3HT: PC_61_BM (1:2) *	* 80 *	* 15 *	* 0.24 *	* 3.0 *
* G1PPT-co-P3HT:PDI-co-Carbazole (2: 1) *	* 50 *	* 2.65 *	* 0.11 *	* 0.021 *
* P3HT:PC_61_BM (2: 1) *	* 510 *	* 7.7 × 10^3^*	* 0.38 *	* 1.5 × 10^3^*

## Data Availability

Data sharing not applicable.

## Supplementary Materials

The following supporting information can be downloaded at: https://www.mdpi.com/article/10.3390/polym14204400/s1, the synthesis and characterization of PDI-co-Carbazole, additional experimental results, and the copies of ^1^H NMR of starting materials and SEM images, Figures S1–S12, Scheme S1–S3 and Table S1.
