# Supplementary material for: Microscopic, Spectroscopic, and Electrochemical Characterization of Novel Semicrystalline Poly(3-hexylthiophene)-Based Dendritic Star Copolymer
Source: Polymers (Basel). 2022 Oct 18;14(20):4400. doi: 10.3390/polym14204400 (PMC9607463; doi:10.3390/polym14204400)
Supplement: Supplementary file 1 [file polymers-14-04400-s001.zip › polymers-1848388-supplementary.pdf]

Microscopic, spectroscopic, and electrochemical characterization of novel semicrystalline poly(3-hexylthiophene)-based dendritic star copolymer

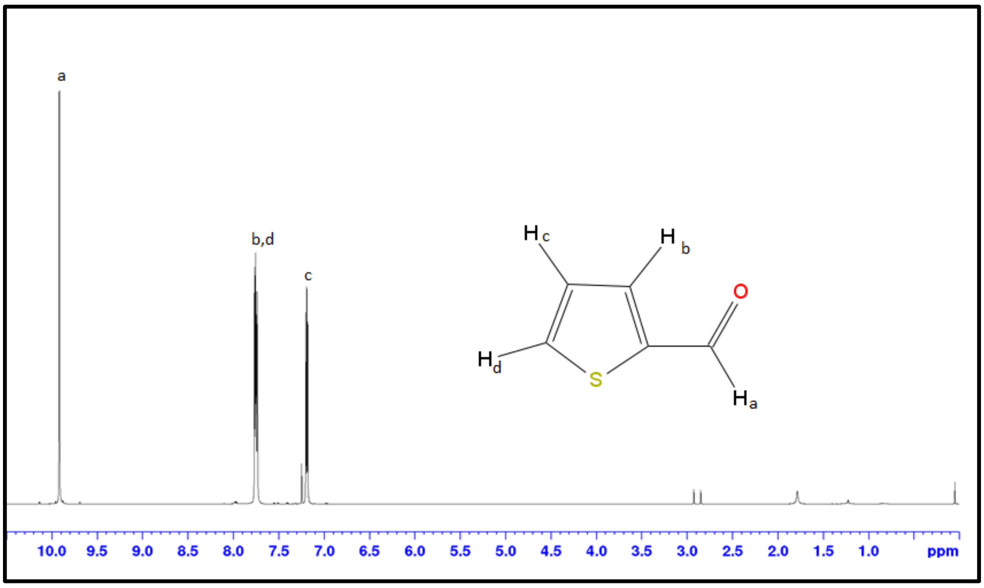

Figure S1 ^1H NMR of 2-thiophene carbaldehyde

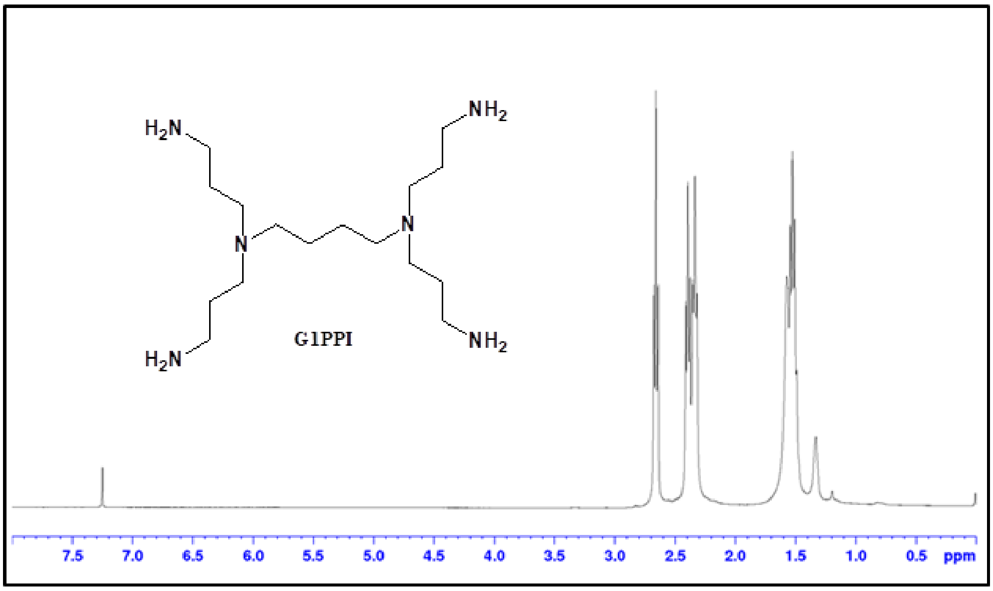

Figure S2. ^1H NMR of generation 1 poly(propyleneimine) tetramine

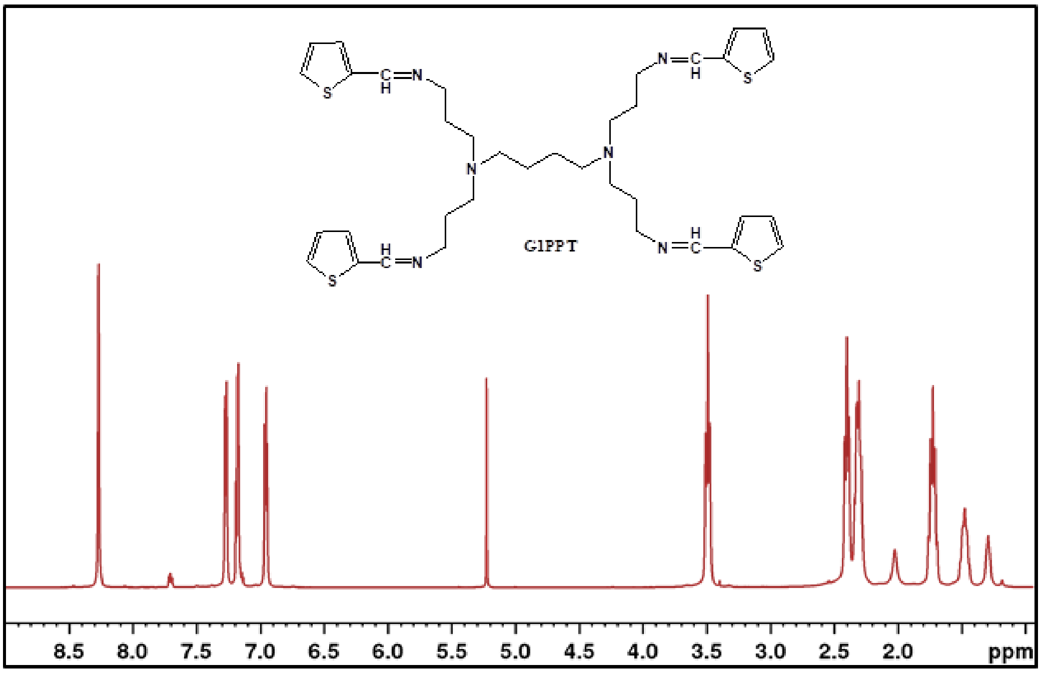

Figure S3. ^{1}H NMR of generation 1 poly(propylene thiophenoimine)
(Intense peak at 5.28 pm is due to CH_3OH)

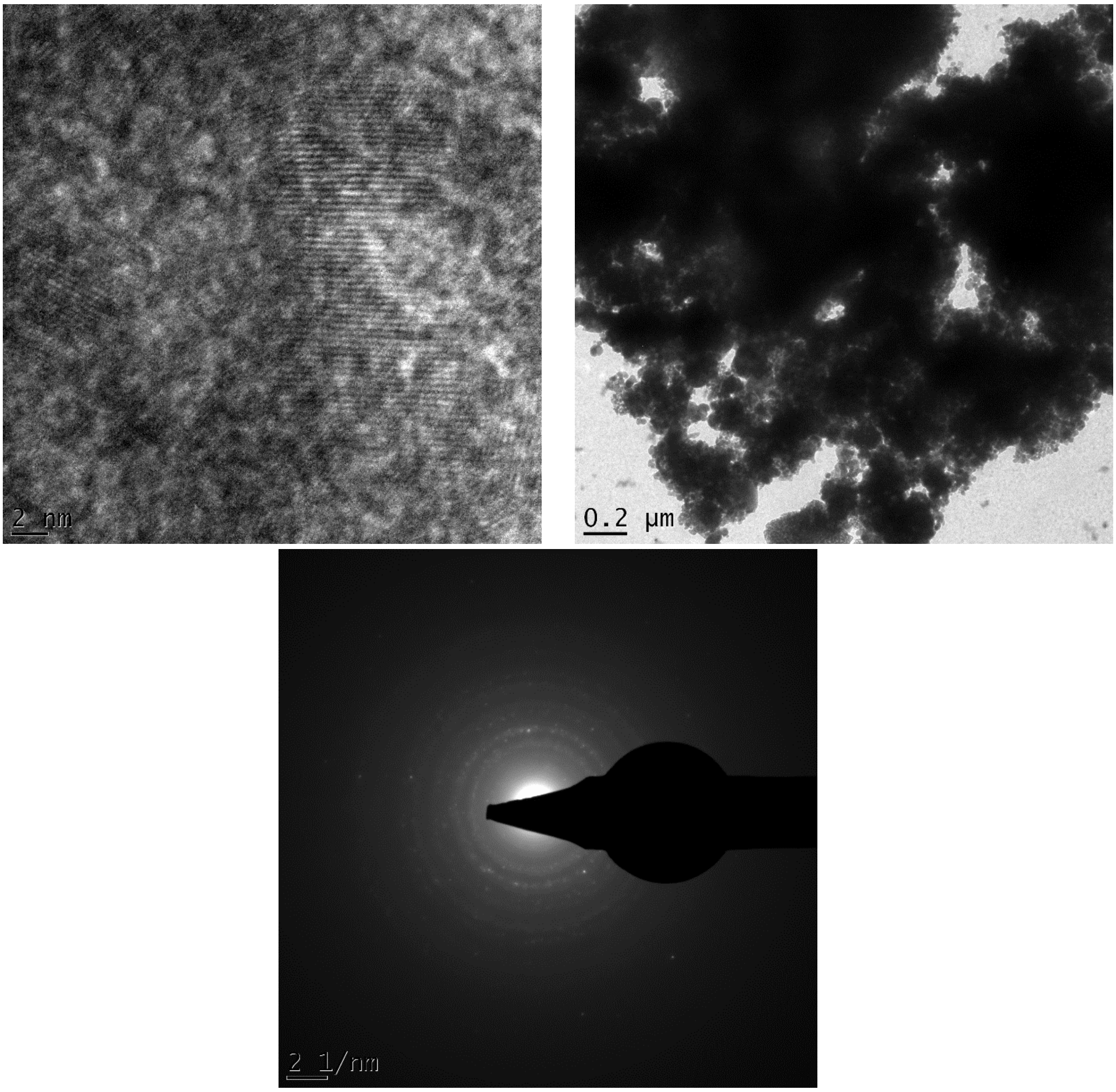

Figure S4 HRTEM and SAED images of G1PPT

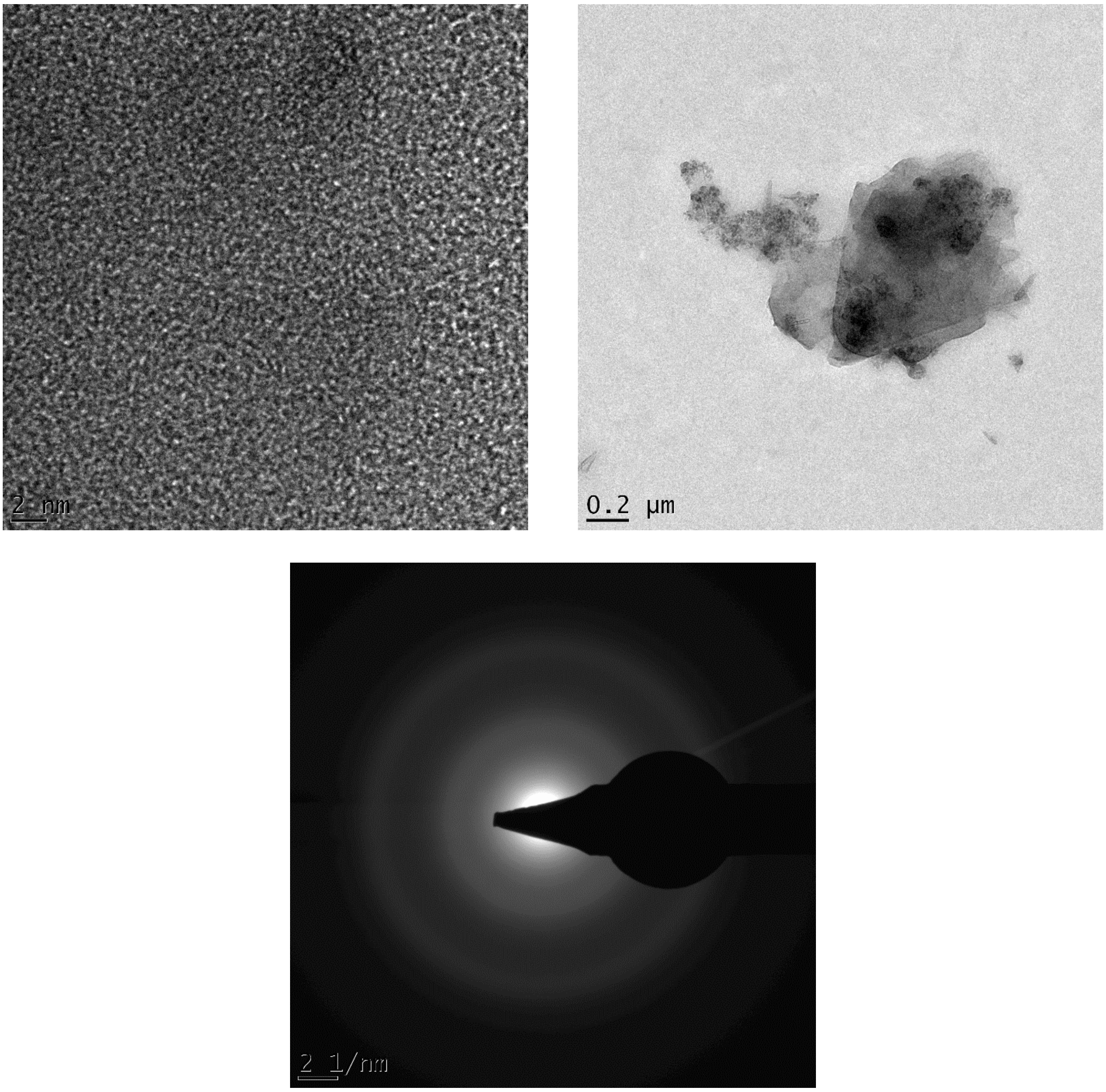

Figure S5. HRTEM and SAED images of 3-HT

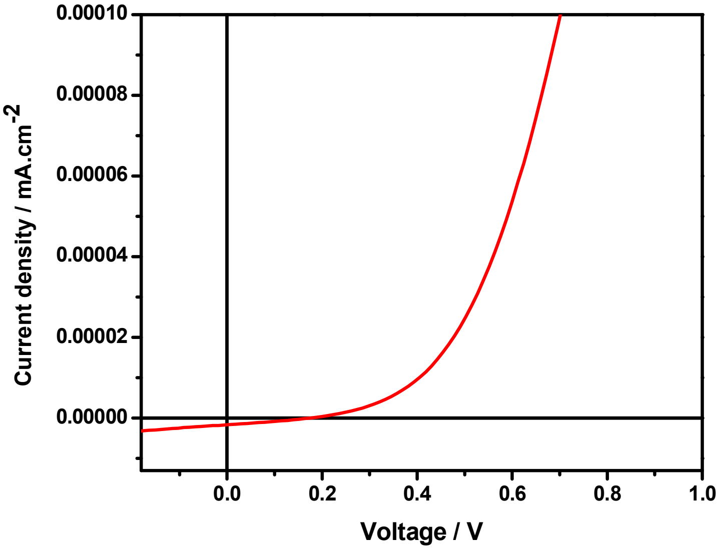

Figure S6: Current -Voltage graph Characteristics of G1PPT-co-P3HT/PDI-co-Carbazole blends

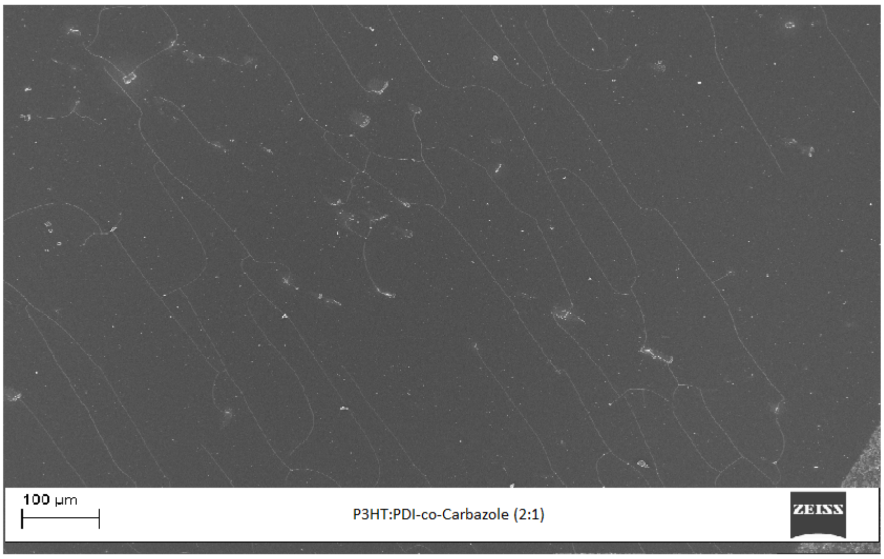

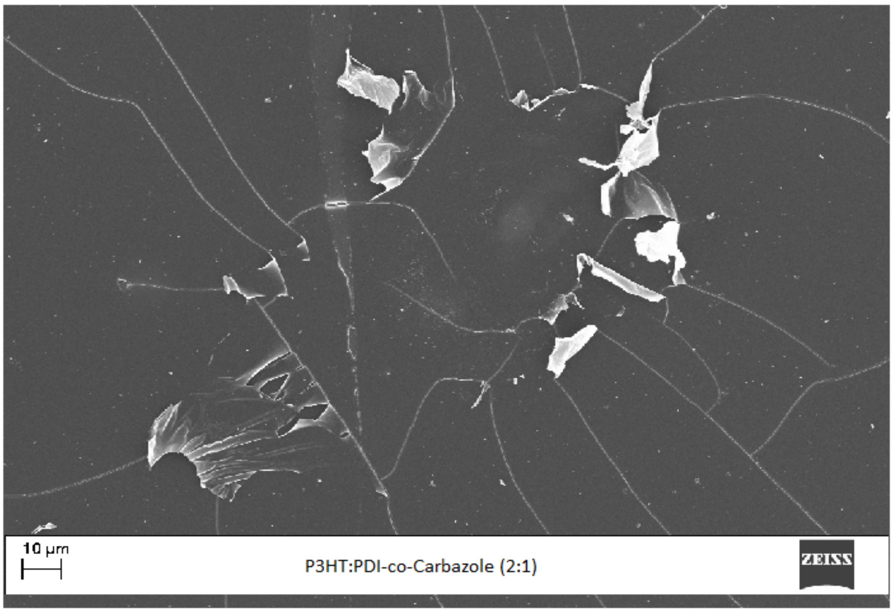

Figure S7. SEM images of P3HT:PDI-co-Carbazole (2:1) on 100 µm and 10 µm scale

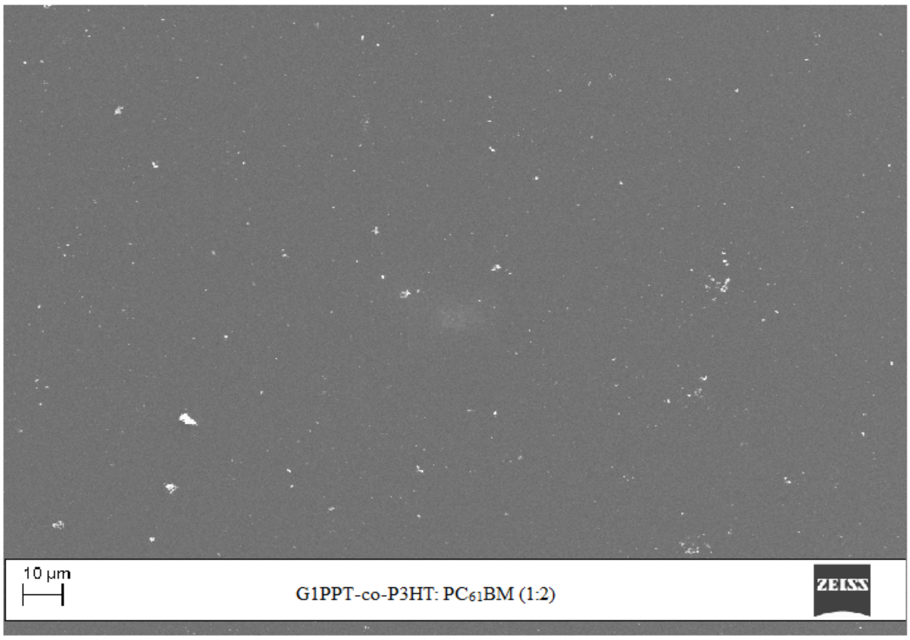

Figure S8. SEM images of G1PPT-co-P3HT:$PC_{61}BM$ (1:2) on 10 µm scale

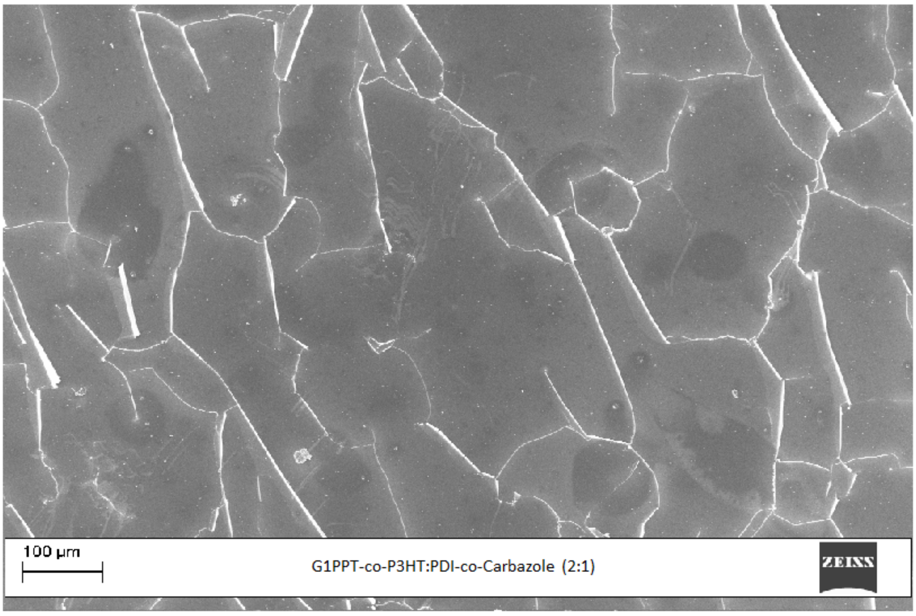

Figure S9. SEM images of G1PT-co-P3HT:PDI-co-Carbazole (2:1) on 100 µm scale

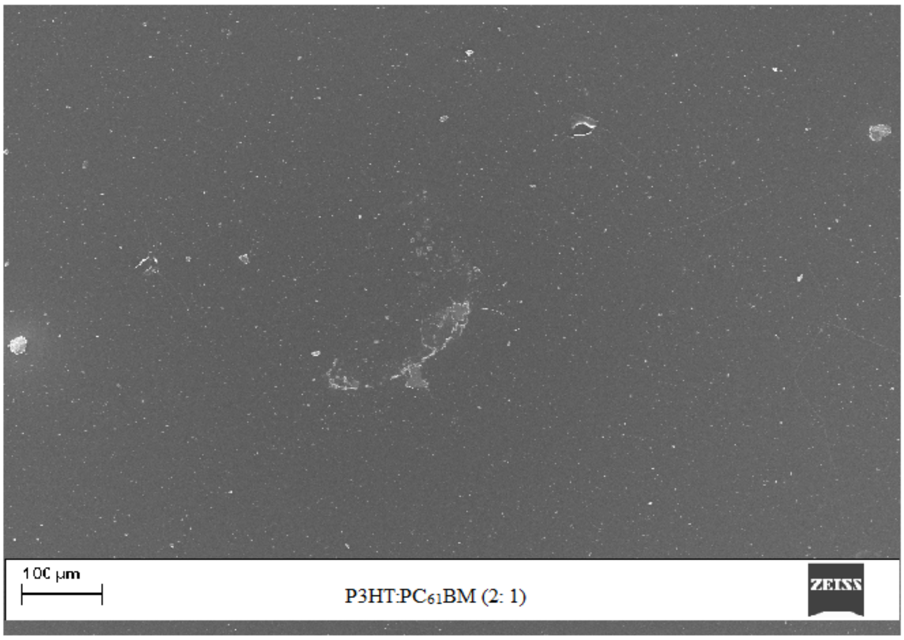

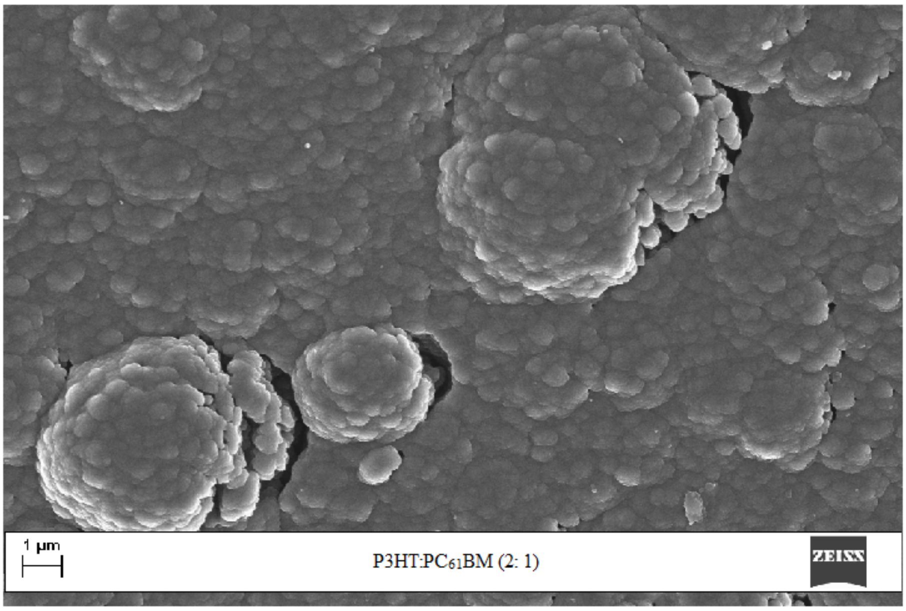

Figure S10. SEM images of P3HT:PC_{61}BM (2:1) on 100 µm and 10 µm scale

1. Synthetic processes for the preparation of poly[N,N'-bis(dodecyl)perylene-3,4,9,10-tetracarboxylic diimide-1,7-diyl-alt-9-(heptadecane-9-yl)carbazole-2,7-diyl] (PDI-co-Carbazole)

1.1 Bromination of perylene-3,4,9,10-tetracarboxyic dianhydride (PTCDA)

Exactly, 7.85 g of perylene-3,4,9,10-tetracarboxylic dianhydride was weighed in a 250 mL three-neck round bottom flask and, 34 mL of 96% sulfuric acid, H_2SO_4 and 29 mL of oleum was added. The mixture was allowed to stir overnight. The catalyst I_2 (0.20 g) was then added. After which the reaction mixture temperature was raised to 85 °C, and bromine (Br_2) solution (2.5 mL) was introduced drop-wise over a period of 8 h. The reaction mixture was then stirred for additional 14 h (while temperature was kept 85 °C). Upon reaction completion, the mixture was left to cool down to room temperature, and Br_2 excess was removed using a gentle Argon (Ar) stream. Addition of 50 mL of water allowed for a precipitate to be formed; which was then filtered through an S3 frit using 30 mL of 70 % and 30 % sulfuric acid and a large amount of water consecutively. Collected sample was dried under vacuum for 48 h.[1] The reaction is depicted in Scheme 3.2.

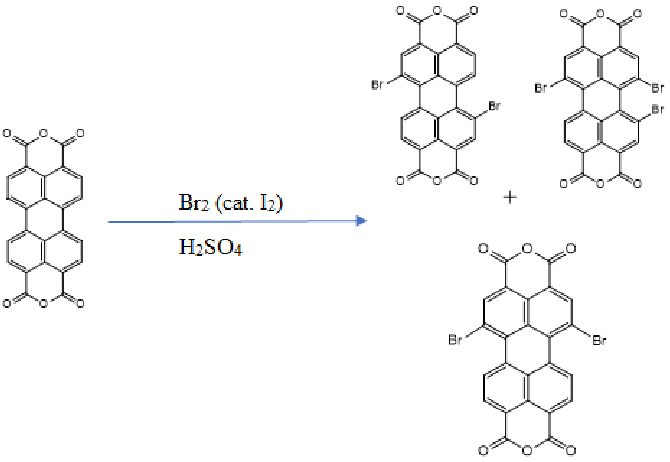

Scheme S1. Bromination of Perylene-3,4,9,10-tetracarboxyic dianhydride

1.2 Imidization of brominated PTCDA

Due to poor solubility of brominated-PTCDA, the material was used as obtained from preceding bromination reaction. 2 g of brominated perylene-3,4,9,10-tetracarboxylic dianhydride was introduced into a three-neck round bottom flask containing 40 mL of N-methyl-2-pyrrolidone (NMP), mixed with 10 mL of acetic acid, and purged under an Ar stream for 15 min. Reaction vessel temperature was raised to 60 °C for 25 min. Two (2) equivalences of N-dodecylamine dissolved in 1 mL of acetic acid were added to the heated mixture and temperature was increased to 120 °C and allowed to stir for 12 h. Upon completion of the reaction, the mixture was poured into 500 mL of water and filtered using professional filter paper with 4 -7 µm pore size and washed three (3) times with methanol. This reaction procedure (Scheme 3.3) is a slight modification of the procedure used by Vajiravelu *et al.*[2]

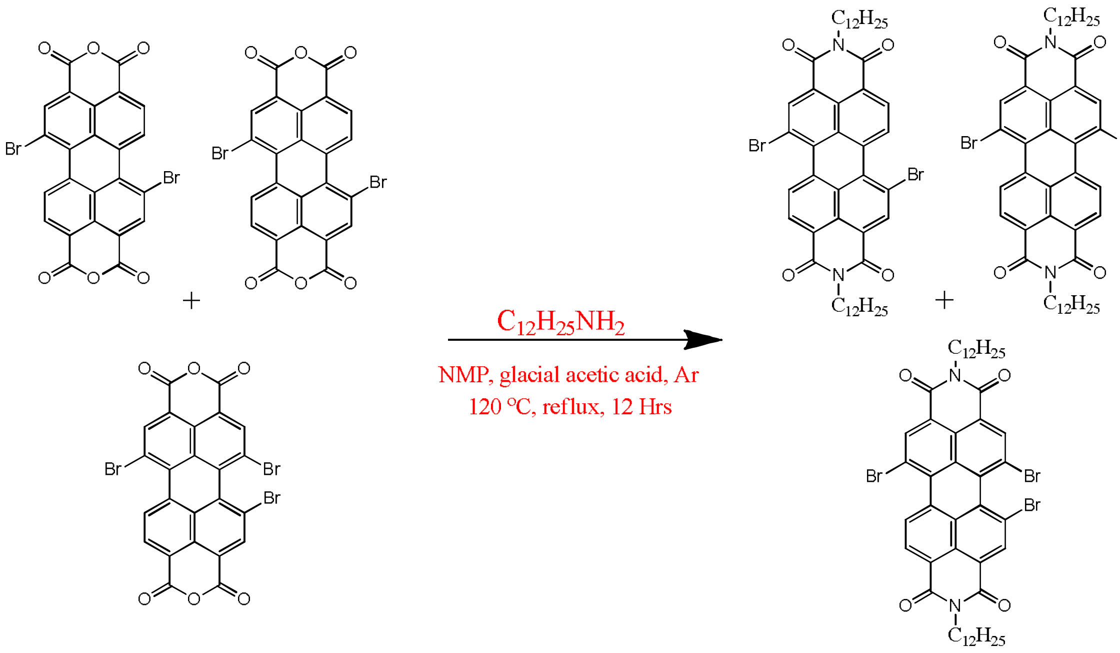

Scheme S2. Imidization of brominated PTCDA

1.3 Suzuki coupling reaction between 1,7-dibromo(N,N'-bis(dodecyl)perylene-3,4,9,10-tetracarboxylic diimide) (PDI-2Br) and 9-(heptadecan-9-yl)-2,7-bis(4,4,5,5-tetramethyl-1,3,2-dioxyborolan-2-yl)-9H-carbazole (Carbazole)

In this work, 1,7-dibromo(N,N'-bis(dodecyl)perylene-3,4,9,10-tetracarboxylic diimide) (PDI-2Br) was copolymerized to 9-(Heptadecan-9-yl)-2,7-bis(4,4,5,5-tetramethyl-1,3,2-dioxyborolan-2-yl)-9H-carbazole (Carbazole), depicted in Scheme 3.4, according to the same synthetic route as reported by Zhou *et al.*[15] Approximately, 570 mg of PDI-2Br was weighed in a completely dried three-neck round bottom flask which was then connected to a condenser and flushed for 1 h under N_2 gas. 425 mg of carbazole was added in 35 mL degassed tetrahydrofuran (THF) and degassed 0.1 M aqueous Na_2CO_3 (30 mL) was also added. The reaction vessel was then allowed to be purged for 30 min to completely remove O_2 in the system. An excess of the catalyst $Pd(PPh_3)_4$ (3 mg, 20% per monomer) was also added in degassed THF and reaction mixture was allowed to stir for 72 h at 110 °C under refllux. End cappings, phenylboronic acid pinacol ester (50 mg) was introduced and left at 110 °C for 1 h; followed by 2-ethylhexylbromide (21 µL) left for 2 h at the same temperature. After cooling, the reaction mixture was transferred into an excess of methanol. The precipitated copolymer was filtered off, washed with water and methanol and dried in the vacuum.

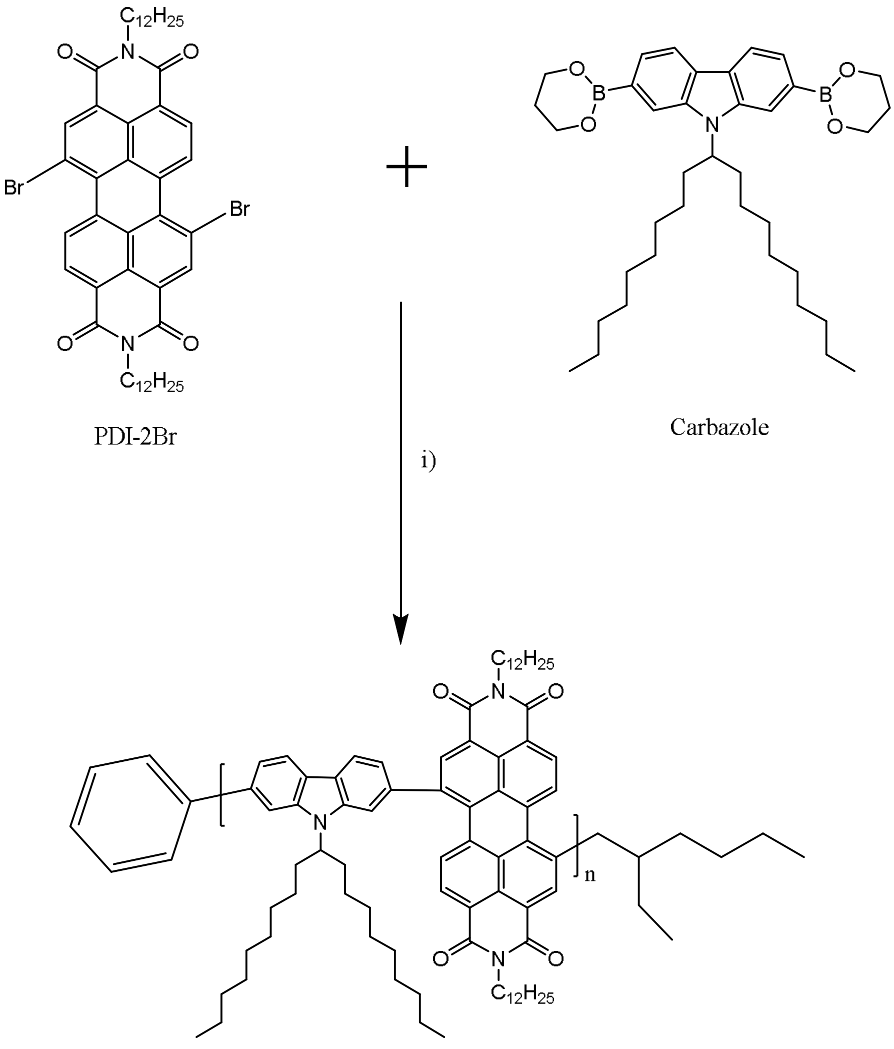

PDI-co-Carbazole

Scheme S3. Synthesis of PDI-co-Carbazole. i) THF, Na_2CO_3 (aq), $Pd(PPh_3)_4$, N_2, 72 h. End cappings: 1) phenylboronic pinacol ester, reflux 1 h, 2) ethylhexylbromide, reflux 2 h.

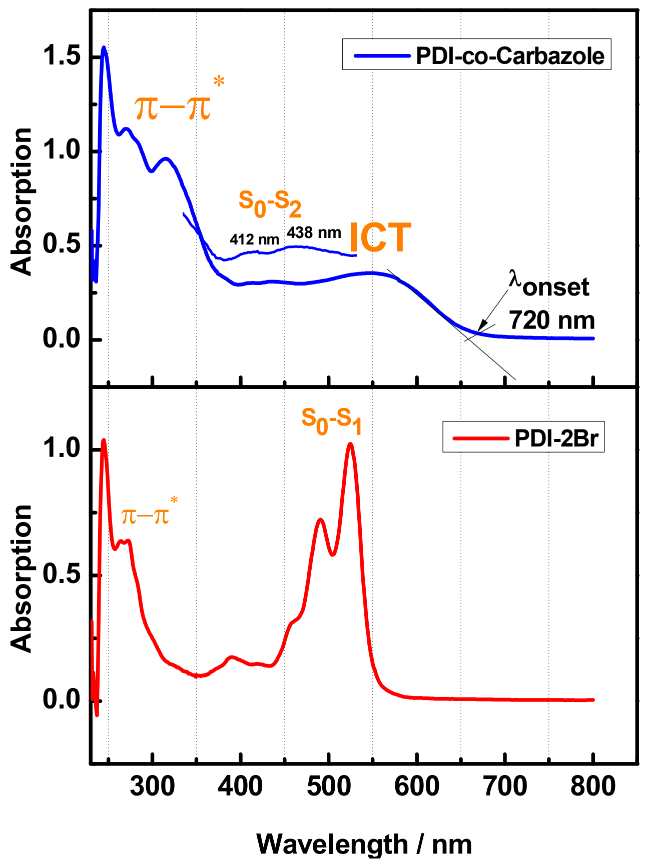

Figure S11. UV-Vis absorption of PDI-2Br and PDI-co-Carbazole in solution

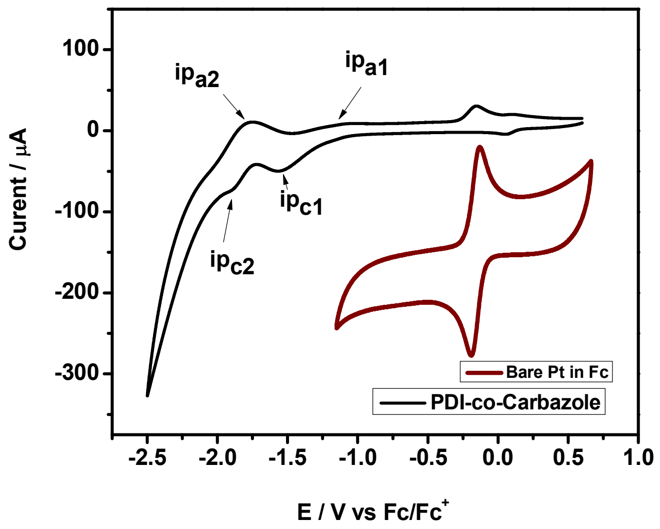

Figure S12. Cyclic voltammogram of PDI-co-Carbazole thin film on Pt electrode 0.1 M Bu_4NPF_6 in acetonitrile at scan rate 50 mV/s versus ferrocene in Ag/Ag^+ reference electrode

Table S1. HOMO, LUMO and band gap E_g^{ec} energy levels of PDI-co-Carbazole

**Materials**	E_{onset}^{ox1} **(V)**	E_{onset}^{red1} **(V)**	E_{IP} **(-E_{HOMO}) (eV)**	E_A **(-E_{LUMO}) (eV)**	E_g^{ec} **(eV)**	E_g^{opt} **(eV)**
PDI-co-Carbazole	0.54	-0.96	5.37	3.87	1.5	1.72
